# Graphene‐Skinned Alumina Fiber: Continuously Scalable Fabrication and Its Electrothermal Application in Fiber‐Reinforced Polymer Composites

**DOI:** 10.1002/advs.202524262

**Published:** 2026-03-04

**Authors:** Xiaobai Wang, Wenjing Jiang, Jingnan Wang, Yuejie Zhao, Enshan Liu, Fushun Liang, Yixin Zhang, Qingqing Liu, Kangyi Zheng, Yuyao Yang, Fan Yang, Xiao Jiang, Yue Qi, Zhongfan Liu

**Affiliations:** ^1^ Department of Chemistry, School of Light Industry Science and Engineering Beijing Technology and Business University Beijing P. R. China; ^2^ Beijing Graphene Institute Beijing P. R. China; ^3^ Academy for Advanced Interdisciplinary Studies Peking University Beijing P. R. China; ^4^ College of Energy, Soochow Institute For Energy and Materials Innovations (SIEMIS), Jiangsu Provincial Key Laboratory for Advanced Carbon Materials and Wearable Energy Technologies Soochow University Suzhou P. R. China; ^5^ Center for Nanochemistry, Beijing Science and Engineering Center For Nanocarbons, Beijing National Laboratory for Molecular Sciences, College of Chemistry and Molecular Engineering Peking University Beijing P. R. China; ^6^ College of Mechanical and Marine Engineering Beibu Gulf University Qinzhou P. R. China

**Keywords:** fiber‐reinforced polymer composite, graphene‐skinned alumina fiber, scalable production, tunable electrothermal performance

## Abstract

Graphene‐skinned alumina fiber (GAF) is a new composite with graphene conformally grown on individual alumina fibers via chemical vapor deposition (CVD). Combining graphene's high conductivity with alumina fiber's strength, GAF shows promising potential for electrothermal applications and structural‐functional integrated composites. Here, it is reported GAF dynamic, continuous, and scalable production using a home‐made roll‐to‐roll (RTR) CVD system, featuring integrated degumming and growth chambers through multi‐stage pressure regulation and nitrogen curtain‐style purging. GAF exhibits excellent flexibility, high tensile strength (>1.1 GPa), and tunable conductivity (40–1260 S m^−1^). The system ensures intra‐/inter‐batch stability with an annual production capacity of 5100–153 000 m, depending on GAF specifications. GAF exhibits superior electrothermal performance, including broad tunable heating temperature (∼620°C in air, >1200°C in vacuum), ultrafast response (>600°C s^−1^), and high cycling stability. Furthermore, GAF presents excellent compatibility with the fabrication process of fiber‐reinforced polymer (FRP) composites. By interlaying GAF between glass fiber/epoxy resin unidirectional (UD) prepregs with different orientations, a GAF‐reinforced polymer (GAFRP) composite is developed, showing adequate resin impregnation and uniform electrothermal heating. These findings highlight GAF's potential for structural‐functional integrated FRP composites, particularly in applications such as electrothermal anti‐/de‐icing of aircraft and wind turbine blades, where FRPs are extensively employed.

## Graphical abstract

Graphene‐skinned alumina fiber (GAF) is a new composite with graphene conformally grown on individual alumina fibers via chemical vapor deposition (CVD). GAF's dynamic, continuous and scalable production is achieved by using a home‐made roll‐to‐roll (RTR) CVD system featuring integrated degumming and growth chambers. GAF possesses tunable conductivity, excellent mechanical performance, and outstanding electrothermal performance, offering potential for electrothermal applications and structural‐functional integrated composites.

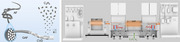

## 1 Introduction

Graphene has attracted great attention because of its ultra‐high electrical conductivity, thermal conductivity, and excellent mechanical strength, and it is considered to be a promising candidate for electrothermal material [1, 2, 3, 4]. The scalable fabrication of high‐quality graphene is the key to promoting its practical application. Chemical vapor deposition (CVD) holds unique advantages in achieving scalable production of high‐quality graphene [5, 6, 7, 8]. However, the graphene grown on traditional metal substrates usually needs to be transferred to the target substrate, which can cause wrinkles, cracks and contamination of graphene [9, 10, 11, 12]. To address this issue, directly growing graphene on the target using substrates is an advantageous strategy, where these substrates are typically dielectric/insulating. This transfer‐free strategy avoids the problems brought by the transfer process and enables continuous and conformal coverage of graphene films on target substrates [5, 13, 14, 15]. Obviously, for the transfer‐free strategy, the chosen target growth substrate will influence the final structure and properties of the graphene‐based composite [16, 17].

Alumina fiber (AF) is a lightweight commercial material with high strength, flexibility, and high‐temperature resistance [18, 19]. Directly conformally growing graphene on individual AFs via CVD can prepare a new composite—graphene‐skinned alumina fiber (GAF), which not only retains AF's excellent lightweight, flexibility and strength but also incorporates graphene's high electrical and thermal conductivity, making it promising for structural‐functional integrated applications in fiber‐reinforced polymer (FRP) composites [20, 21]. For traditional FRP composites, the main pursuit is structural properties. However, the development of functional FRP composites has now become a current forefront trend. Thermoset serves as the primary matrix material in FRPs. The typical manufacturing process involves laminating prepregs, followed by curing using vacuum bagging [22]. Unidirectional (UD) prepreg is a highly anisotropic material. With its flexibility in layup design, it can optimize the mechanical performance of the FRPs according to the primary load direction and achieve lightweighting [23, 24, 25]. Glass fiber is widely used as a reinforcement in FRPs due to its high strength and excellent toughness. Given GAF's structural and compositional similarity to glass fiber, GAF exhibits excellent compatibility with the existing FRPs fabrication process. Moreover, owing to its potential electrothermal properties, GAF can be strategically interlaid between glass fiber/epoxy resin UD prepregs as a functional fiber, providing customized pathways for heating. And the orientation and density of GAF can be precisely designed according to specific functional requirements, such as uniform heating or directional thermal management. The integration of glass fiber UD prepreg and GAF can result in a structural‐functional integrated FRP composite, which is lightweight, high‐strength, and capable of fulfilling the GAF's electrothermal task. It is expected to offer potential application scenarios in the anti‐/de‐icing of aircraft, automobiles and wind‐turbine blades, where FRPs are commonplace [26, 27].

The scalable production of GAF is a prerequisite for meeting the demands of scalable practical applications [8, 28, 29]. Currently, unlike traditional flat substrates used for CVD growth of graphene, scalable graphene deposition on fiber‐structured substrates has yet to be explored. The dynamic roll‐to‐roll (RTR) strategy may represent a reliable approach for the scalable preparation of continuous GAF [30]. Although RTR growth of graphene has been achieved on metal foils, continuous RTR growth of graphene on non‐catalytic fiber substrates remains unexplored. This calls for customized design of equipment structures and process optimization specifically tailored to non‐metallic fiber substrates. For example, how to achieve precise tension control of the fibers without compromising their structural integrity. It is worth noting that AF typically undergoes degumming to remove its surface polymer layer. Therefore, establishing a connection between the degumming and growth chambers while maintaining strict atmospheric isolation remains highly challenging.

In this study, GAF's dynamic, continuous and scalable production was achieved by using a home‐made RTR CVD system featuring integrated degumming and growth chambers. This RTR system employs a tension control module, a transport deviation correction module and a feed‐back module. It achieves comprehensive and precise control over the fiber tension, transmission path and unwinding/rewinding rate (0–500 mm min^−1^), enabling the integrity of the fiber structure during high‐speed production. By adopting multi‐stage pressure regulation and nitrogen curtain‐style purging, the atmosphere of the two chambers was isolated, successfully overcoming the challenges brought by the integrated design. Computational fluid dynamics (CFD) simulations confirm that the mole fraction of air in the growth chamber was extremely low, and the temperature and pressure distribution were uniform. These simulations fully verified the rationality of this design. Furthermore, an exploration of the growth process in GAF's scalable production was conducted. The GAF demonstrates tunable graphene thickness and conductivity, and it also exhibits intra‐/inter‐batch stability. Moreover, GAF demonstrates high tensile strength (>1.1 GPa) and outstanding electrothermal performance. It presents broad tunable heating temperature (∼620°C in air and >1200°C in vacuum), ultrafast response (>600°C s^−1^) and high cycling stability. Meanwhile, a GAF‐reinforced polymer (GAFRP) composite was fabricated by interlaying GAF between the glass fiber UD prepregs with different orientations. GAF exhibits excellent compatibility with the manufacturing process of FRP composites, and adequate resin impregnation was achieved. GAF serves as an embedded functional component due to its excellent electrothermal properties, enabling the GAFRP composite to realize uniform and tunable heating temperature, making it an advanced structural‐functional integrated FRP composite. GAF exhibits a broad application potential in FRP composites and electrothermal fields, especially in electrothermal anti/de‐icing systems of aircraft and wind turbine blades [31].

## 2 Results and Discussion

### 2.1 CVD Growth of Graphene on AF

**FIGURE 1:**
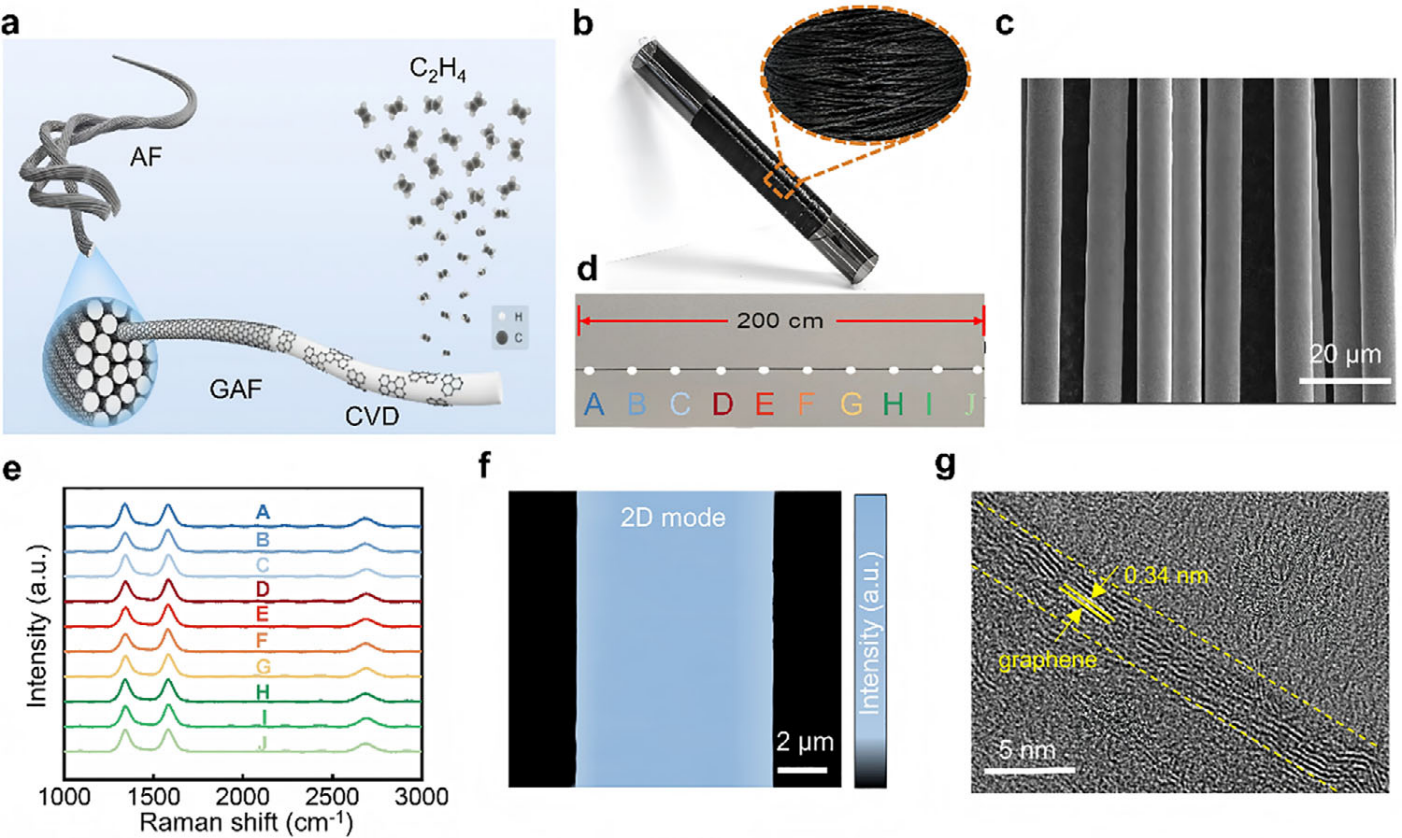
CVD growth of graphene on AF. (a) Schematic diagram of graphene CVD growth on AF using ethylene as the carbon precursor. (b) Photograph of GAF (∼50 m in length). (c) SEM image of GAF with well‐maintained fiber profile (the fiber diameter is ∼9 µm). (d) Photograph of a GAF sample with a length of ∼2 m. (e) Raman spectra (normalized to G peak intensity) of GAF collected at points (A‐J) in (d), spaced ∼22 cm apart along the central axis of the GAF. (f) Intensity mapping of the Raman 2D peak (∼2700 cm^−1^) of a graphene ribbon obtained by etching the core AF of GAF. (g) Cross‐sectional HR‐TEM image of graphene on AF.

Figure 1a illustrates the schematic diagram of graphene CVD growth on twisted AF. When growing graphene on catalyst‐free substrates via CVD, the decomposition of the carbon precursor is dominated by thermal pyrolysis. The carbon source is decomposed to generate active carbon species, which are subsequently adsorbed and diffused across the substrate surface, leading to the formation of continuous graphene films [14]. In this study, ethene was chosen as the carbon‐source precursor due to its low decomposition energy barrier. Using the commercially available AF (Figure S1) and self‐developed dynamic, continuous and scalable production system of GAF (Figure S3), the CVD growth was conducted at a temperature of ∼1100°C to enable the efficient carbon precursor pyrolysis and the uniform deposition of continuous graphene film on each fiber surface. As shown in Figure 1b, a continuous GAF 50 m in length can be successfully rolled up, demonstrating excellent flexibility. The scanning electron microscopy (SEM) image in Figure 1c shows that the fiber morphology is well‐preserved after CVD growth, with graphene uniformly coated on each fiber surface. To assess the uniformity of as‐grown graphene on the AF surface, multi‐point Raman spectroscopy measurements were conducted on GAF. As illustrated in Figure 1d, the measurement points were spaced ~22 cm apart and marked with different colors. The corresponding Raman spectra (Figure 1e) demonstrate nearly identical Raman signal profiles at every point, indicating the excellent coverage uniformity of graphene on AF surfaces. The Raman 2D peak mapping of an individual graphene ribbon obtained by etching the core AF of GAF further indicates continuous and uniform coverage of graphene films on each fiber surface (Figure 1f). The thickness of the graphene film was directly measured by atomic force microscopy (AFM). The measured thickness is twice the actual thickness of the grown graphene plus 0.8 (the value of 0.8 serves as a correction, see more detail in Figure S2). As shown in Figure S2, the graphene ribbon exhibits a uniform thickness of ∼6.27 nm, corresponding to ∼8 layers of graphene. The cross‐sectional high‐resolution transmission electron microscopy (HR‐TEM) images of GAF further confirmed that the continuous and ∼8‐layer structure of the graphene with a lattice fringe spacing of ∼0.34 nm (Figure 1g).

### 2.2 Dynamic, Continuous, and Scalable Production System of GAF

The scalable preparation of GAF not only meets the growing demand for practical scale applications but also enhances growth efficiency. The RTR process offers the key advantages of continuity and automation [32]. Although the RTR process enabled continuous graphene growth on metal foils, extending it to a catalyst‐free fiber substrate AF requires customized equipment design and process parameter optimization. such as the precise control of AF tension and transmission path under high‐temperature, dynamic conditions. In addition, since the surface polymer layer of AF can affect the quality and uniformity of graphene layers during the CVD growth, the polymer layer must be completely removed before GAF's production. Therefore, to achieve efficient and continuous RTR dynamic CVD growth on AF, the integrated design of degumming and the growth chambers is of vital importance.

**FIGURE 2:**
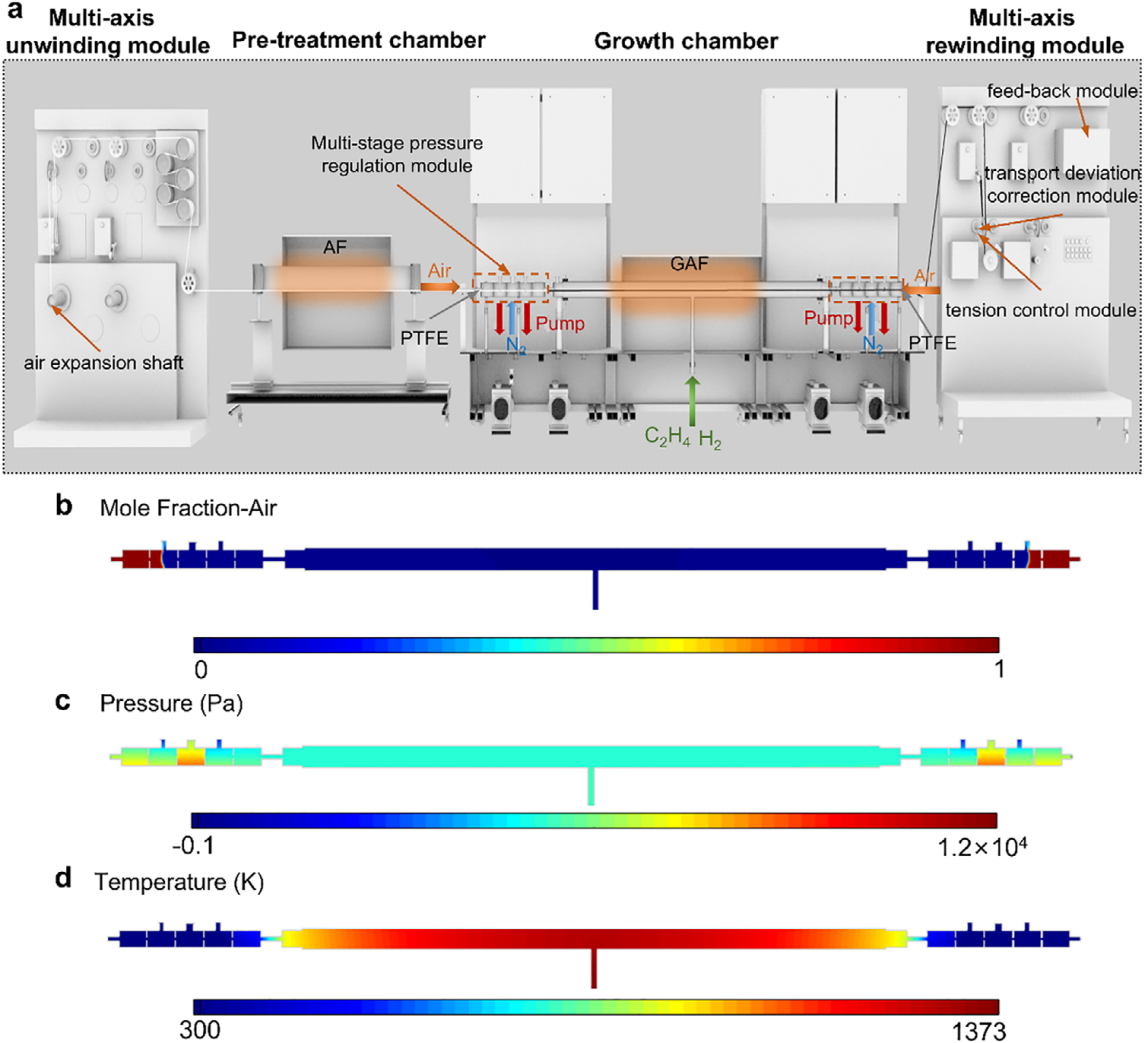
Dynamic, continuous, and scalable production system of GAF. (a) Schematic diagram of GAF's dynamic, continuous, and scalable production system, which primarily comprises a multi‐axis unwinding module, pre‐treatment chamber, multi‐stage pressure regulation modules, growth chamber and a multi‐axis rewinding module. (b–d) CFD simulations for the distribution of mole fraction of air (b), pressure distribution (c), and temperature distribution (d) in the growth chamber.

As shown in Figure 2a, we independently developed a dynamic, continuous, and scalable production system of GAF. This system adopts a home‐made RTR CVD system and integrates both degumming and growth chambers. In this system, AF was smoothly transported through multi‐axis unwinding modules, a pre‐treatment chamber, and a multi‐stage pressure regulation module into the growth chamber at the set unwinding rate (0–500 mm min^−1^) to achieve high‐temperature graphene deposition. Meanwhile, the multi‐axis rewinding module is responsible for rewinding the graphene‐skinned AF after growth through multiple guide rollers at a controlled rewinding rate (0–500 mm min^−1^). Moreover, the RTR CVD system is equipped with a tension control module, which enables stable tension during the AF transmission, thereby preventing damage to the fiber structure. The process is also equipped with an automatic transport deviation correction module and a feed‐back module. These ensure accurate running paths of the fibers, guarantee real‐time monitoring and precise control of the rewinding and unwinding rates, and maintain their stability.

Notably, an integrated design of the degumming and growth chambers was developed. This design minimizes the risk of damage and contamination that may be caused by manual transfer and other operations, and improves production efficiency [33, 34]. However, when conducting integrated design, the aerobic environment required by the degumming chamber fundamentally conflicts with the oxygen‐free environment required by the growth chamber. When oxygen infiltrates the growth chamber at a high temperature of ∼1100°C, oxygen can trigger a vigorous exothermic reaction with hydrogen, causing deflagration, and can restrain hydrogen's reduction reaction, resulting in graphene defects and a decline in overall film quality [35]. Additionally, the presence of oxygen can react with the carbon precursor to form CO_2_ or CO, thereby depleting the supply of active carbon atoms and leading to a reduced graphene growth rate or even complete cessation of growth. Consequently, it is essential to ensure the effective isolation of the atmospheres in the two chambers. To prevent oxygen from infiltrating the growth chamber, a multi‐stage pressure regulation and introduction of inert nitrogen is employed between the two chambers. The multi‐stage pressure regulation module gradually reduces the partial pressure of oxygen, progressively based on the differential pumping principle, while nitrogen is introduced in a curtain‐style purging to form a dynamic gas‐lock barrier. The collision‐scattering interactions between nitrogen and oxygen molecules further impede oxygen infiltration [36, 37]. This design can effectively maintain the oxygen concentration in the transitional zones on both sides of the growth chamber within ∼1% (Figure S4), while simultaneously sustaining the growth chamber pressure at ∼60 torr. The resulting low‐pressure growth environment enhances the mass transfer rate of active carbon species and promotes uniform concentration distribution across the growth chamber, thereby improving the graphene film thickness uniformity and suppressing the formation of amorphous carbon on AF [8, 38, 39]. Meanwhile, to further block the pathways of oxygen infiltration, a polytetrafluoroethylene (PTFE) gasket (aperture: ∼1 mm, Figure S5) and four layers of baffles (aperture: ∼5 mm) are added to the transition zones on both sides of the growth chamber to establish a physical barrier to gas infiltration. To verify the effectiveness of the designs, a multi‐physics‐coupled analysis was conducted using CFD simulations to model gas flow fields, including oxygen mole fraction distribution and pressure distributions. As shown in Figure 2b, the implementation of the multi‐stage pressure regulation modules and the nitrogen purging resulted in an extremely low mole fraction of air in the growth chamber. Moreover, the multi‐stage pressure regulation ensures that the growth chamber maintains a uniform pressure distribution (Figure 2c), thereby ensuring the uniformity and controllability of graphene deposition on the AF.

For the growth chamber, the overall length is ∼200 cm. It is designed to enable the deposition of graphene film on a continuously moving, pre‐treated AF under a controlled atmosphere comprising H_2_, C_2_H_4_, etc. Given that AF exhibits minimal catalytic activity toward the CVD growth of graphene, to prepare qualified GAF, it is essential to promote the full pyrolysis of the carbon precursors at high temperature (above ∼1000°C) to generate the requisite activated carbon species [40]. During operation, the system must keep all positions of the moving AF across a consistent flow field and thermal field, which is critical for achieving uniform graphene coverage and ensuring structural continuity of the grown graphene film [20]. In this regard, higher requirements are imposed on the growth chamber's constant temperature zone. To address this, the isothermal zone of the chamber has been extended to ∼900 mm. Additionally, thermal insulation materials are applied to the chamber walls to minimize the heat loss in the chamber, thereby promoting the formation of a uniform thermal and gas flow field within the chamber. The thermal field simulation result (Figure 2d) further confirms the uniform temperature field distribution within the growth chamber. Notably, the design of a three‐port quartz tube structure has been adopted, featuring a centrally located gas inlet and two gas outlets at both ends. This design prevents external oxygen from mixing into the reaction atmosphere. The central gas inlet is ingeniously placed at the bottom of the high‐temperature zone, which is particularly effective in promoting uniform gas distribution and enhancing reaction efficiency. When the carbon source enters through the lower gas inlet, the extremely high temperature at the bottom of the high‐temperature zone provides sufficient thermal energy to fully crack the carbon precursors, thus minimizing the formation of undesired by‐products. The resulting active carbon species are subsequently carried upward by the ascending gas flow and uniformly adsorb onto the AF surface. Additionally, the exhaust ports at both ends of the chamber establish a bidirectional exhaust path, effectively preventing concentration gradients that may arise from unilateral exhaust and promoting the uniform distribution of the reactive gases in the growth region. At the same time, the upward gas flow carrying the by‐products generated by the carbon source is rapidly evacuated through the two end exhaust ports by pumping from both ends, thus reducing the likelihood of by‐product deposition on the AF surface and improving the overall quality of the graphene film. The simulation results of the distribution of mole fraction of gas (Figure S6) indicate that the mole fractions of the carbon source and hydrogen are uniformly distributed throughout the reaction chamber, and the mole fraction on the chamber wall is the same as that in the bulk, which is conducive to the formation of continuous and uniform graphene films on AF.

### 2.3 Scalable Preparation of GAF

**FIGURE 3:**
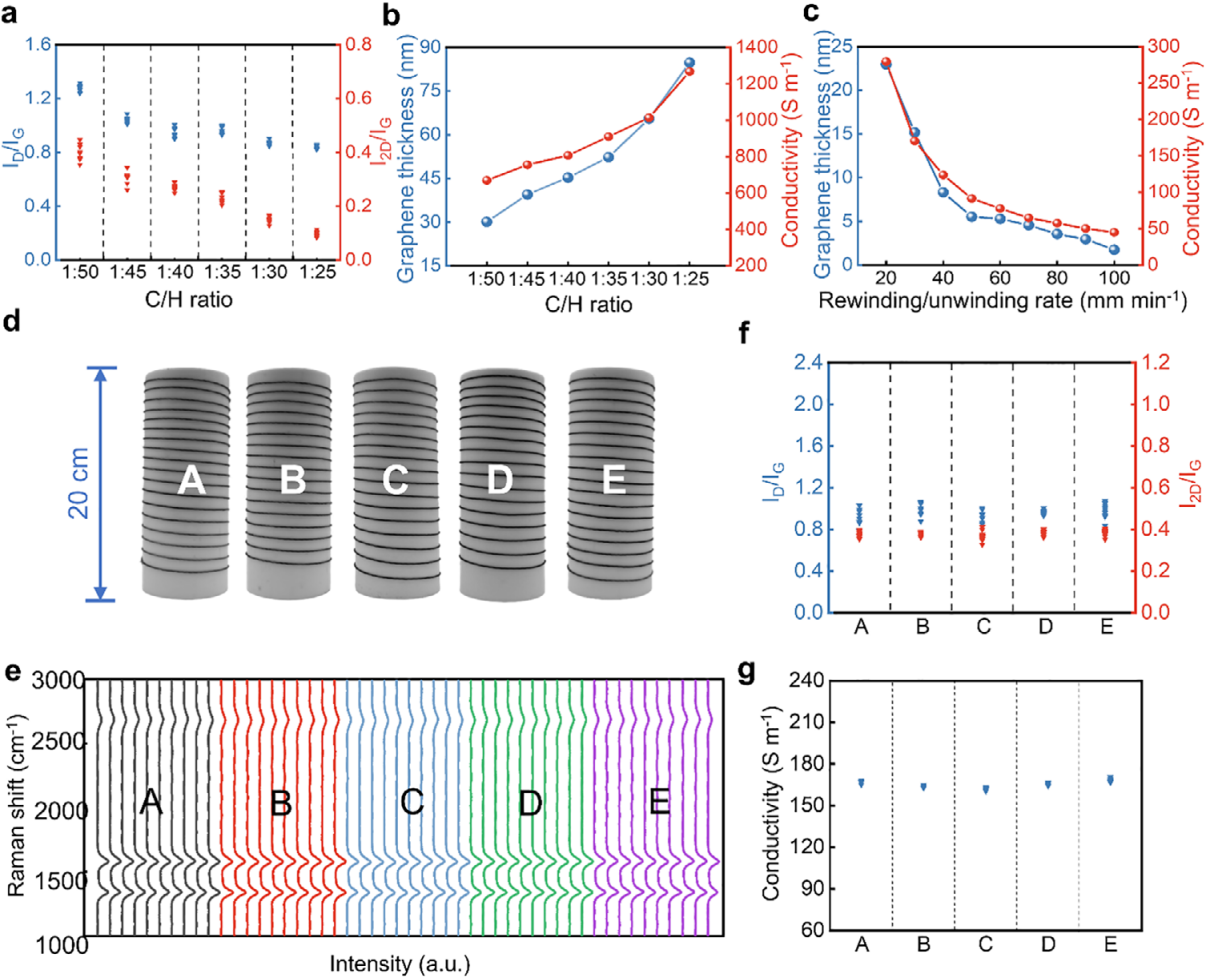
Scalable preparation of GAF. (a) Raman peaks intensity ratios *I*_D_ */I*_G_ and *I*_2D_ */I*_G_ of GAF obtained at different C/H ratios (1:25, 1:30, 1:35, 1:40, 1:45, 1:50). (b) Graphene thickness and conductivity of GAF obtained with different C/H ratios. (c) Graphene thickness and conductivity of GAF obtained at different rewinding/unwinding rates (10–100 mm min^−1^). (d) Photograph of GAF (with a length of ∼90 cm) from 5 batches (A–E), each under identical growth conditions. (e) Raman spectra collected at 10 evenly spaced positions, with uniform intervals of ∼10 cm over a lateral distance of ∼90 cm along the central axis of GAFs from the 5 batches in (d). (f) Raman intensity ratios *I*_D_ */I*_G_ and *I*_2D_ */I*_G_ derived from the Raman spectra in (e). (g) Conductivity collected between two adjacent sampling points, yielding 9 evenly spaced intervals, with uniform intervals of ∼10 cm over a lateral distance of ∼90 cm along the central axis of GAF from the 5 batches in (d).

After developing a dynamic, continuous, scalable production system of GAF, it became crucial to explore the graphene growth conditions on AF substrates. Such exploration enables the optimization of the quality of grown graphene and modulates its thickness [41]. As shown in Figure 3a,b and Figure S7, an increase in the C/H ratio leads to a decrease in both *I*_D_ */I*_G_ and *I*_2D_ */I*_G_, suggesting better graphene crystalline quality and thicker graphene layer thickness, respectively. Meanwhile, the conductivity of GAF is highly dependent on the thickness of the grown graphene. By modulating the thickness, the conductivity can be regulated, which will cater to a variety of application scenarios with diverse requirements for conductivity. The conductivity of the GAF increases as thickness increases and becomes tunable within the range of ∼660 to ∼1260 S m^−1^ (Figure 3b). Figure 3c and Figure S8 show that as the rewinding/unwinding rate increases, corresponding to a shorter growth time of graphene in the high‐temperature CVD chamber, the graphene thickness gradually decreases, leading to a gradual decrease in GAF's conductivity. Tables S1 and S2 show a more quantitative correlation between the growth conditions (C/H ratio and rewinding/unwinding rate), thickness of graphene, graphene layers, and conductivity of GAF. Therefore, GAF demonstrates tunable graphene thickness and tunable conductivity.

The inter‐batch stability of GAF's preparation process is crucial for the mass production of GAF. Figure 3d shows the photograph of GAF samples from five batches (A‐E), demonstrating consistent surface contrast among batches. Figure 3e shows the corresponding Raman spectra of GAFs (collected every ∼10 cm at equal intervals along each batch of GAF, with 10 measurements per batch and 90 cm length in total). It indicates that the Raman signals of GAFs remain highly consistent between batches and within batches. As illustrated in Figure 3f, the calculated *I_D_/I_G_* and *I_2D_/I_G_* exhibit approximately identical values in the Raman spectra of GAFs from five batches (A‐E), indicating consistent graphene crystalline quality and layer thickness, respectively. As shown in Figure 3g, Conductivity measurements of GAF exhibit its intra‐ and inter‐batch consistency and stability. As shown in Figure S9 and Table S3, further demonstrates the excellent intra‐batch and inter‐batch consistency and reproducibility of GAF in crystalline quality and layer thickness of graphene and conductivity of GAF. In summary, the GAF prepared using our self‐developed GAF's dynamic, continuous, and scalable production system demonstrates GAF's excellent inter‐batch stability and repeatability. And this system can achieve an annual production capacity of 5100–153 000 m based on the GAF specifications (see more details for the calculation of GAF's annual production capacity in Note S2).

### 2.4 Mechanical and Electrothermal Performance of GAF

**FIGURE 4:**
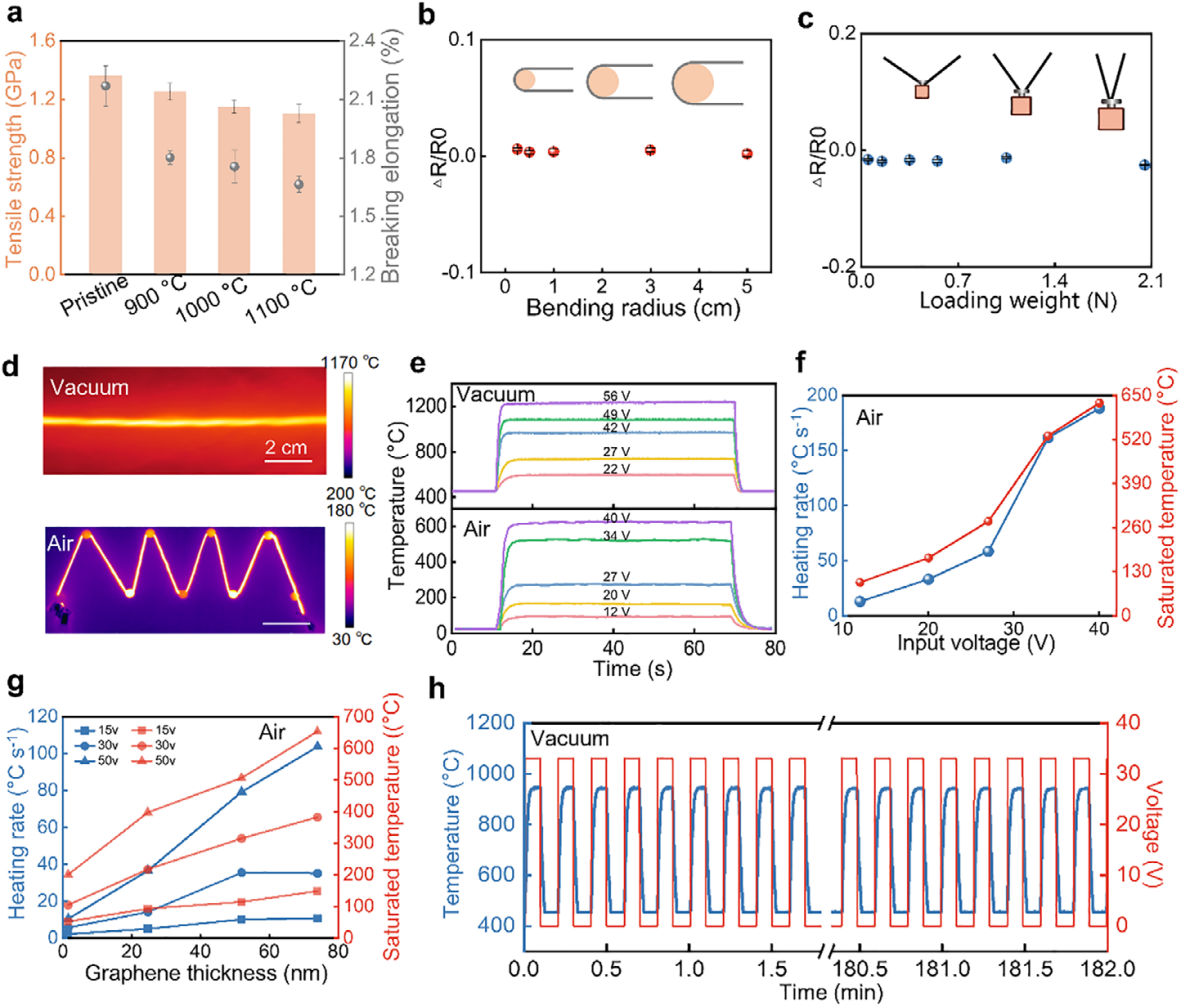
Mechanical and electrothermal performance of GAF. (a) Tensile strength and breaking elongation of pristine AF and GAF obtained at different growth temperatures (∼900°C, ∼1000°C, ∼1100°C for ∼2 h). The error bars represent the standard deviations (*n* = 5). (b) Resistance variation of GAF subjected to bending deformations at various bending radii (0.25–5 cm), with 100 bending times applied at each radius. The error bars represent the standard deviation (*n* = 5). (c) Resistance variation of GAF under various loading weights (0.05–2.05 N). The error bar represents the standard deviation (*n* = 5). (d) Infrared thermal image of GAF heating wire in both air and vacuum environments (air: 165.2 ± 5.1°C, 25 cm, ∼1040 S m^−1^, ∼18 V voltage input; vacuum: 1158.8 ± 19.1°C, 10 cm, ∼1040 S m^−1^, ∼52 V voltage input). (e) Temperature profiles of GAF heating wire under different input voltages in an air and a vacuum environment. (f) Heating rate and saturated temperature of GAF heating wire under different input voltages in an air environment. (g) Effect of graphene thickness of GAF heating wire on the heating rate and saturated temperature under different input voltages (∼15 V, ∼20 V, ∼50 V) in an air environment (the length of GAFs is all 10 cm, the conductivities of GAF are ∼43, ∼304, ∼907 and ∼1107 S m^−1^, respectively). (h) Temperature response of GAF heating wire under square wave voltage cycles in a vacuum environment (0–33 V, a period of 12 s, 910 continuous cycles).

It can be seen from Figure 4a that AF exhibits a high tensile strength of up to ∼1.36 GPa, and the tensile strength of GAF decreases slightly with increasing growth temperature (see more detail in Figures S10 and S11). Tensile strength measurement across different batches (A‐E) shows negligible variation (Figure S12), indicating the inter‐batch consistency of mechanical performance. As shown in Figure 4b,c, GAF shows strong mechanical deformation tolerance. Within a bending radius range of ∼0.25 to ∼5 cm, the resistance of the GAF remains highly stable, demonstrating excellent durability under bending conditions. Under applied loads ranging from ∼0.05 to ∼2.05 N, the relative resistance change (ΔR/R) of GAF remains below ∼2.5%, indicating the strong pressure resistance of GAF. These results suggest a strong bonding force or robust interfacial adhesion between graphene and the AF substrates, preventing sliding and detachment of the graphene film during mechanical stress.

Graphene is uniformly and continuously covered on AF, endowing the fibers with excellent conductivity, thus achieving electrothermal performance. Meanwhile, the fibers retain the lightweight and high flexibility of AF. Based on this, a Joule heating wire was fabricated to evaluate the electrothermal performance of GAF. As shown in Figure 4d, infrared thermal images of GAF heating wire in both air and vacuum environments display a uniform temperature distribution (air: 165.2 ± 5.1°C; vacuum: 1158.8 ± 19.1°C), along with good mechanical flexibility. In addition, it can be seen from Figure 4e that the temperature profile of the GAF heating wire at various input voltages in an air environment. The GAF heating wire rapidly reaches its corresponding saturated temperature (T_S_) within seconds, and T_S_ exhibits a broad tunable range depending on the different applied input voltages. The maximum Ts reaches up to ∼620°C. As shown in Figure 4f, the GAF heating wire exhibits a high electrothermal response, or heating rate (defined as the increasing rate of temperature within the time it takes to reach 90% of the T_S_), which is primarily attributed to the low density, ordered thermal conduction pathways and minimal interfacial heat loss inherent in the structure of GAF. For example, we can see that the heating rate reaches ∼188.24°C s^−1^ at an input voltage of ∼40 V. Notably, comparisons of the saturated temperatures and heating rates of five batches (A‐E) of GAF in an air environment (Figure S13a) reveal the stable electrothermal performance across batches. In addition, the detailed effects of graphene thickness and input voltage on both T_S_ and heating rate of GAF heating wire in an air environment are shown in Figure 4g. The conductivity of GAF heating wire is correlated with the layer thickness of graphene (Figure S14), and the conductivity affects the input power at a given input voltage, thus affecting the saturated temperature. Therefore, the heating capacity of GAF heating wire can be tuned via the graphene layer thickness. As demonstrated in Figure 4g, both Ts and the heating rate of GAF heating wire can be adjusted by the graphene thickness and input voltage. GAF heating wire with thicker graphene thickness at the same input voltage or GAF heating wire with higher input voltage at the same graphene thickness showed faster heating rate and higher saturation temperature. As shown in Figure S15, the GAF heating wire was subjected to a 0–30 V square‐wave voltage with a period of 16 s for 607 cycles in an air environment. Its saturated temperature remained constant, while the temperature response was highly synchronized with the input voltage, demonstrating the GAF's excellent cycling stability.

In an air environment, graphene begins to be oxidized at a temperature above ∼500°C, which weakens the thermal stability of graphene. In contrast, under the vacuum condition, the structure of graphene remains stable at temperatures up to ∼2800 K [42, 43]. Notably, numerous electrothermal applications are conducted in vacuum, such as vacuum coating processes and vacuum heat treatment processes of optical devices and electronic components, and precise thermal processing of metals [44]. Figure 4e and Figure S16 show that the saturated temperature and heating rate of GAF heating wire in vacuum are strongly dependent on the applied input voltages. The maximum observed saturated temperature of the GAF heating wire reaches as high as ∼1240°C, accomplished by an exceptionally rapid heating rate. For example, at an input voltage of ∼56 V, the heating rate reached ∼683.7°C s^−1^ (see more details for the high‐temperature heating test apparatus under vacuum in Figure S17). Similarly, under vacuum, the five batches (A‐E) of GAF exhibit similar saturated temperatures and heating rates under different input voltages, indicating GAF's inter‐batch electrothermal stability in a vacuum environment (Figure S13b). As shown in Figure S18, the thicker graphene thickness on the GAF heating wire at the same voltage results in both higher saturated temperatures and faster heating rates. Figure 4h shows the thermal cycling performance of GAF under vacuum. When the GAF heating wire is subject to a square‐wave voltage input between 0 and 33 V with a period of 12 s, the temperature profiles remain unchanged over 910 cycles, exhibiting its excellent cycling stability.

### 2.5 Preparation and Electrothermal Performance of GAFRP Composite

**FIGURE 5:**
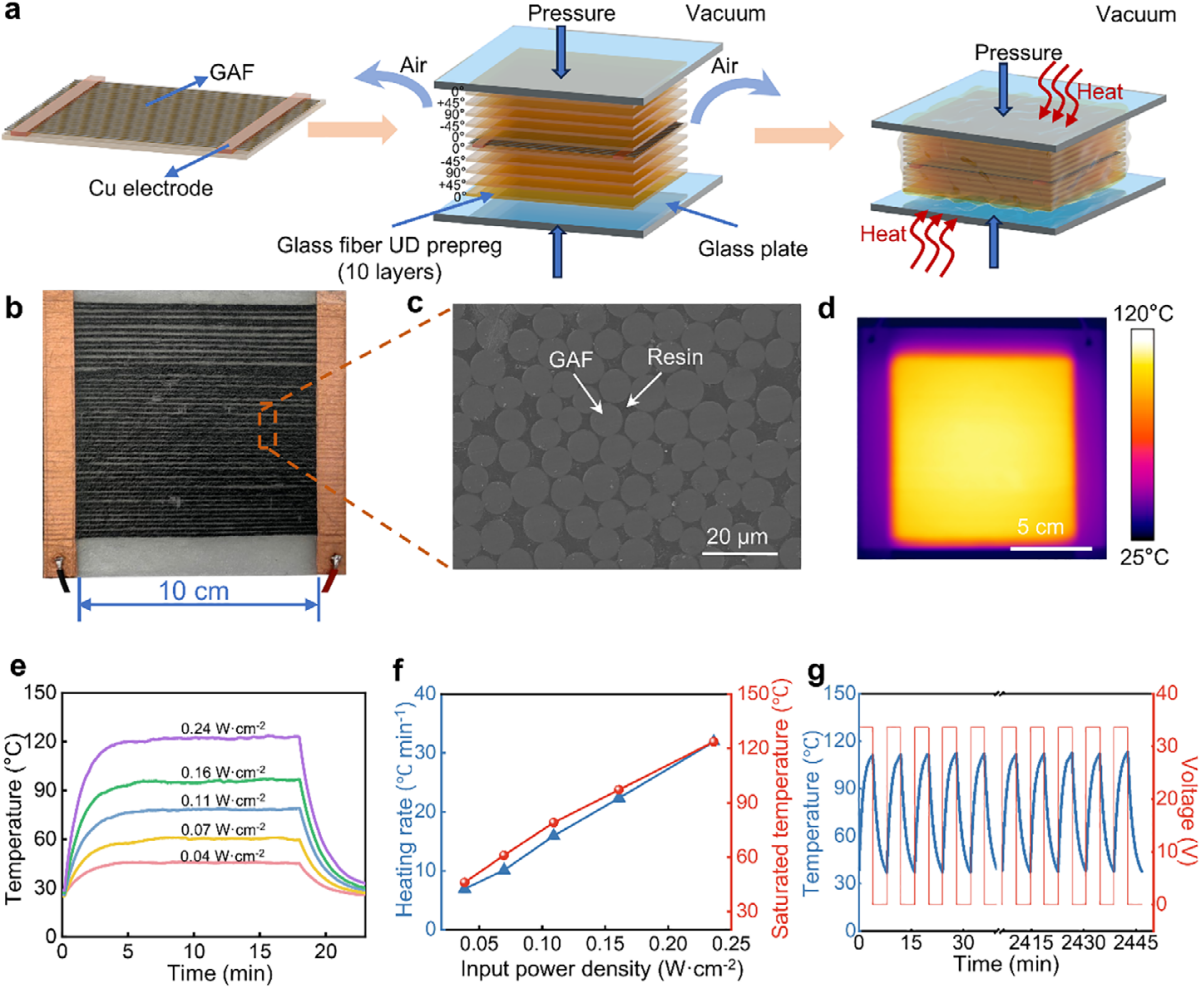
Preparation and electrothermal performance of GAFRP composite. (a) Schematic illustration of the manufacturing process of the GAFRP composite, comprising four stages: lay‐up of 10‐layer glass fiber UD prepreg (orientation: 0°/+45°/90°/‐45°/0), placement of GAF (each length of 12 cm, fiber laying density of ∼0.5 per cm^2^, fiber spacing of 2 mm; each conductivity of ∼1260 S m^−1^), vacuum degassing and thermal curing. (b) Photograph of the produced GAFRP composite. c) Cross‐sectional SEM micrograph of the GAFRP composite. (d) Infrared thermal image of GAFRP heating plate (110 ± 0.7°C, ∼36.5 V voltage input). (e) Temperature profiles of the GAFRP heating plate under different input power densities. (f) Heating rate and saturated temperature of the GAFRP heating plate under different input power densities. (g) Temperature curve of GAFRP heating plate under a square wave from 0 to 33.6 V, with a period of 8 min and 306 cycles.

Composite based on UD prepreg is characterized by exceptional directional mechanical properties and designability. It enables the maximum strength and modulus in the fiber orientation. Based on this, according to the actual load‐bearing conditions of the composite, the structure can be designed by precise layup of UD prepreg at specific angles, achieving optimal mechanical performance and ultimate weight reduction. Glass fiber is widely used as reinforcement in FRP composites due to its high strength and excellent toughness. By arranging continuous glass fibers in parallel and immersing them in partially cured resin (e.g., epoxy), a thin and adhesive glass fiber UD prepreg is formed. Given that GAF exhibits structural and compositional similarities with glass fiber, it possesses perfect process compatibility with the manufacturing of FRPs. Additionally, due to its excellent electrothermal performance, GAF can be used as a functional fiber in FRPs, providing customizable heating paths, resulting in a structural‐functional integrated GAFRP heating plate. Figure 5a shows the preparation procedure of the GAFRP composite. Using a quasi‐isotropic layup method, the layup orientation of glass fiber UD prepreg plies was set to 0°/+45°/90°/‐45°/0, while the intermediate layer places the parallel and non‐interwoven GAFs (tow laying density: 0.5 per cm^2^; tow spacing: 2 mm), and copper electrodes were affixed on both sides of the GAFs. Following the vacuum degassing stage, entrapped air was removed, resulting in an 11‐layer GAF/UD prepreg composite laminate. Subsequent thermal curing fully crosslinked the resin and produced the final GAFRP composite. The composite exhibited quasi‐isotropic behavior, with uniform in‐plane strength and stiffness, thereby mitigating premature failure under undirectional loading. Figure 5b shows the photograph of the fabricated GAFRP composite, which displays a flat surface without distortion or warping. The embedded GAFs within the resin remain continuous, straight, and uniformly aligned without bending or twisting.

The wettability between fibers and resins is evaluated by resin droplet spreading over the fibers and contact angle measurement. As shown in Figures S19 and S20, the resin shows better spreading and wetting ability on the GAF surface in comparison with AF. The cross‐sectional SEM image (Figure 5c) of the sample reveals a homogeneous fiber distribution, along with smooth, dense, pore‐free, and uniform resin regions. The resin thoroughly infiltrates and encapsulates the GAF, ensuring strong interfacial bonding between the GAF and the resin without any pores and a uniform microstructure. It can be seen from Figure S21a,b that the AFRP composite and GAFRP composite exhibit a high interlayer shear strength of up to ∼64 MPa and ∼62 MPa, indicating the incorporation of graphene does not exert an effect on the interlayer shear properties. Figure S21c shows the SEM cross‐section morphologies of AFRP and GAFRP composite after interlaminar shear failure, respectively. After the interlayer shear test, gradual slippage and crack formation occurred between fibers and resin in the prepreg layer, yet no crack occurred near AF or GAF in the middle layer, indicating strong interfacial bonding in these regions. Figure S22 shows the tensile stress‐strain curves and tensile strength of AFRP and GAFRP. The maximum stress or tensile strength of both exceeded 500 MPa.

Figure 5d presents the infrared thermal image of a GAFRP heating plate (10 cm × 10 cm), revealing a uniform temperature distribution (110 ± 0.7°C). GAF itself exhibits excellent heating uniformity. The arrangement of parallel and non‐interwoven GAF forms an efficient thermal conduction network throughout the entire material, while the resin matrix functions as a buffer or a homogenizer by filling between the highly thermally conductive fibers, slowing down excessive heat transfer along any single direction and promoting more uniform heat diffusion in all directions. Consequently, the composite is less susceptible to localized hot spots and steep temperature gradients. Figure 5e shows the surface temperature of the GAFRP heating plate under different power densities, demonstrating a broad tunable range (∼46.0–∼123.7°C). Figure 5f shows that both the heating rate and saturated temperature of the GAFRP heating plate scale positively with the input power density, and the plate exhibits a rapid thermal response. When the input power density is ∼0.24 W cm^−2^, the heating rate reaches ∼31.9°C min^−1^. This performance arises from the intrinsic rapid electrothermal response of the GAF, which enables the temperature of the entire plate to rise quickly. Additionally, the cyclic stability of the GAFRP heating plate was evaluated. Over 306 square wave voltage cycles (0–33.6 V, 8 min period), the panel displayed a highly reproducible temperature response with consistent peak values, evidencing great stability (Figure 5g). Furthermore, the electrothermal performance of GAFRP under a simulated convective cooling environment showed that the GAFRP reached the target steady‐state surface temperature (5°C) within only 150 s when the ambient temperature was −20°C and the wind speed was 16 m s^−1^, demonstrating a high electrothermal conversion efficiency. The required steady‐state power can be adjusted according to different application scenarios (Figure S23). Overall, the outstanding electrothermal performance of the GAFRP heating plate makes it an excellent thermal management functional material, combining the benefits of structure and function, and is expected to offer strong potential for applications in scenarios such as electrothermal de‐icing and anti‐icing.

## 3 Conclusions

In this work, we developed a dynamic, continuous, and scalable production system of GAF to achieve the scalable production of GAF. This system employs a home‐made RTR CVD system and an integrated design of the degumming and CVD growth chambers by incorporating multi‐stage pressure regulation and implementing nitrogen curtain‐style purging. Through an in‐depth exploration of the scalable production process of GAF, optimization of the quality of graphene grown on AF has been achieved. GAF exhibits tunable thickness of graphene and conductivity. In addition, GAF features intra‐/inter‐batch stability, laying a robust foundation for the continuous and stable GAF production. Meanwhile, the prepared GAF features excellent mechanical performances such as high tensile strength, excellent bending resistance and strong pressure resistance, as well as excellent electrothermal performance. It demonstrates uniform temperature distribution along with good flexibility, a broad tunable heating temperature range and ultrafast response in both air and vacuum environments. Moreover, GAF exhibits high process compatibility with the FRP composite manufacturing. A GAFRP composite was fabricated by integrating GAF with glass fiber UD prepreg, which exhibited adequate resin impregnation capability and excellent electrothermal performance. GAF will become a promising candidate for multifunctional applications in advanced FRP composites.

## 4 Experimental Section

### 4.1 CVD Growth of GAF

AF (F‐72 type) used for CVD growth of graphene is sourced from Rongrong New Materials Company, China. Before the graphene deposition, AF was loaded into a self‐developed mass production CVD system. The specific loading procedures are described as follows:

The three‐temperature zone tube‐type degumming furnace and tube‐type growth furnace were simultaneously activated. The degumming chamber was heated to the desired temperature of ∼800°C, and the air was pumped into the chamber to remove the polymer layer from the AF surface. Meanwhile, the growth chamber was heated to a temperature range between ∼900 and ∼1100°C. Then, both ends of the growth chamber were pumped down to a pressure of 1000 Pa, and a continuous nitrogen flow of 20000 sccm was introduced to purge residual air and maintain an inert atmosphere. For the graphene growth process, the fiber unwinding and rewinding rate was set to 10–100 mm min^−1^.

When the degummed AF moved to the growth chamber, a controlled gas flow of ethene (5–10 sccm) and hydrogen (250–500 sccm) was introduced into the growth chamber. Upon completion of the growth, the ethene and hydrogen flows were terminated, and the heating of the two chambers was turned off. In 10 min, the nitrogen flow was ceased and the vacuum pumps were shut down. The detailed growth conditions of GAF were provided in Note S3.

### 4.2 Transfer Process

GAF was first cut into small pieces and immersed in a mixed etching solution composed of 50% HF solution and deionized water at a 1:1 volume ratio for several hours to remove the inner alumina fiber core. Then the GAF was collapsed into freestanding graphene ribbons, which were subsequently rinsed three times with deionized water. Finally, the graphene ribbons were transferred onto a silicon wafer and dried under an infrared heat lamp. The resulting samples were prepared for subsequent thickness characterization of graphene films.

### 4.3 Preparation of GAFRP Composite

The medium‐temperature low‐density glass fiber/epoxy resin UD prepreg was purchased from Shanghai Huibai New Materials Technology Co., LTD. (fiber areal weight: ∼200 g m^2^; resin content: ∼38%; cured ply thickness: ∼0.1 mm; glass transition temperature: ∼140°C). The single‐layer glass fiber UD prepreg was cut into 10 sheets of 120 mm × 120 mm. Five plies of prepreg were first laid in the orientations of 0°, +45°, 90°, −45°and 0°, respectively. Copper meshes were then affixed on both sides to serve as electrodes, with copper foil tabs attached to the ends of the electrodes to allow drilling for copper wire connections. GAFs were subsequently interlaid on the prepregs (tow laying density: 0.5 per cm^2^; tow spacing: 2 mm), copper tapes were stuck on both sides, and another five plies of prepreg were further laid in the symmetrical sequence direction (0°, −45°, 90°, +45°, 0°).

The prepared prepreg laminate was positioned on a glass plate coated with release agent, followed by the placement of auxiliary materials, including breather felt, isolation film, vacuum bag and vacuum nozzle. Vacuum compaction was performed to eliminate entrapped air between the layers, after which the assembly was cured in an oven. The curing cycle involved heating to 120°C at 2°C min^−1^, holding for 60 min, and then cooling to 60°C at 1.5°C min^−1^, during which the pressure was maintained at 0.5 MPa.

After curing, the laminate was demolded. Finally, the edges of the sample were polished with sandpaper, and holes were drilled at the copper foil position to connect copper wires. The resulting GAFRP composite was thus obtained and used as a heating plate.

### 4.4 Characterizations

The prepared samples were characterized using Raman spectroscopy (Horiba, LabRAM HR‐800, laser excitation at a wavelength of 532 nm), SEM (FEI Quattro S, operated at an acceleration voltage of 10 kV), HR‐TEM (JEOL, JEM 2100F, operated at an acceleration voltage of 200 kV) and AFM (Bruker Dimension Icon with tapping mode).

### 4.5 Conductivity Test

Copper tapes were affixed on both sides of GAF to function as the electrodes and connected to a digital multimeter (Keithley 2400, Tektronix, dual‐tip probe, contact resistance compensation< 0.1%) to measure the bulk resistance (R). The conductivity (G) of GAF was calculated according to the following formula:

$$\;G=\frac{1}{\rho }=\frac{L}{\mathrm{RS}}$$

where G is the conductivity (S·m^−1^), R is the measured bulk resistance (Ω), ρ is the resistivity, L is the length of the GAF (m), and S is the cross‐sectional area of the GAF (m^2^). The cross‐sectional area S can be calculated by dividing the linear mass density (0.0041 g cm^−1^) by the material density of a single fiber (2.91 g cm^−3^) (see more details for the calculation method of GAF conductivity in Note S4).

### 4.6 Tensile Strength Measurement

The tensile strength of GAF was tested using an electronic universal material testing machine (MTS E43.104) (Figure S10a). The specific testing procedure was as follows: an auxiliary board (A4 paper) sized 20 mm × 50 mm was prepared, with a 10 mm × 30 mm slot cut in its center area (left of Figure S10b). GAF samples each were randomly selected from different growth temperatures (Conditions: ∼900°C, ∼1000°C, ∼1100°C for 10 sccm C_2_H_4_, 500 sccm H_2_ and rewinding/unwinding rate of ∼10 mm min^−1^) or batches (Conditions: ∼1100°C for 10 sccm C_2_H_4_, 500 sccm H_2_, and rewinding/unwinding rate of ∼30 mm min^−1^). Each sample was 50 mm in length and was placed vertically along the longitudinal axis of the center slot on the auxiliary board. AB epoxy glue was applied to both ends of each fiber sample to adhere them to the board (Figure S10c and the middle of Figure S10b). After curing at room temperature for 24 h, the samples were vertically fixed between the machine's upper and lower fixtures, aligning their central axis with the tensile direction (Figure S10d). Before applying the load, both sides of the auxiliary paper board were cut (Figure S10e and right of Figure S10b).

Then the tensile speed was set to 10 mm min^−1^ on the control panel of the machine. After cutting both sides of the auxiliary paper board, the machine was activated and stretched the sample at the set speed until fiber fracture, recording the instantaneous tensile force (F). For each temperature condition and each batch of GAF, the measurement was repeated five times. The maximum tensile strength (σ) was calculated according to the following formula:

$$\sigma =\frac{F}{a}$$

where *σ* is the tensile strength (GPa), F is the breaking load (N), and a is the cross‐sectional area of the GAF (mm^2^), which can be calculated by dividing the linear density (0.004 g cm^−1^) by the material density of a single fiber (2.91 g cm^−3^).

### 4.7 Bending Test and Loading Weight Test

To evaluate the mechanical stability of the GAF, GAF samples (with ∼20 nm of graphene thickness) were randomly cut to a length of 35 cm. Two copper electrodes were affixed to both ends of the GAF and connected to a digital multimeter (Keithley 2400, Tektronix) to continuously monitor changes in the GAF's bulk resistance. For the Bending test, GAF was bent repeatedly 100 times around metal rods with different radii (ranging from 0.25 to 5 cm), and the resistance was recorded after each bending cycle. For the Loading Weight test, standard calibration weights (ranging from 10 to 100 g) are gradually suspended at the midpoint of the GAF to apply a vertical mechanical load, and the resistance of the GAF is measured in real‐time during the application of each load. Measurements were performed in five replicates for each bending radius and loading weight.

### 4.8 Electrothermal Performance Test

The experiments to evaluate the electrothermal performance of GAF were conducted under both atmospheric pressure and vacuum conditions. After applying voltage to the electrodes at both ends of GAF with a direct current (DC) power supply, the infrared images of GAF were captured in real time using an infrared camera (Fluke Ti 10). Both the heating and cooling curves were recorded to assess the dynamic thermal response of the GAF.

### 4.9 CFD Simulation

The geometric models of the growth chamber and the multi‐stage pressure regulation modules on both sides as an integral were constructed by using the Space Claim Direct Modeler (SCDM) module in ANSYS software. The dimensions in the geometric models were the same as the actual dimensions. The growth chamber is ∼200 cm in length and adopts a three‐port quartz tube structure (a middle gas inlet, and gas outlet outlets (inner diameter ∼80 mm) at both ends). Two gaskets were added to both ends of the integrated device, and a hole was drilled at its central position (aperture: ∼1 mm) to allow the AF to smoothly pass through the growth chamber. In addition, four layers of baffles (aperture: ∼5 mm) are added to each of the multi‐stage pressure regulation modules. The integrated device includes 1 mixed gas inlet of C_2_H_4_ and H_2_ (diameter:24 mm), 2 gas inlets of N_2_ (diameter:26 mm), 2 leakage ports of air (diameter:1 mm) and 4 pump outlets (diameter:17 mm).

Based on the Bernoulli equation, the ideal gas state equation theory, and the proportion of mass fraction, the import and export gas flow rates are calculated. The temperature field, pressure distribution and gas flow field of the model, such as the gas molar fraction distribution, were simulated by CFD using ANSYS Fluent software. The boundary conditions were set according to the actual experiments of CVD [38]. The inlet and outlet boundary conditions are set with boundaries of velocity, pressure and temperature. (see more details for the boundary conditions, solver setting and mesh partitioning in Note S1).

## Conflicts of Interest

The authors declare no conflicts of interest.

## Supporting information




**Supporting File**: advs74712‐sup‐0001‐SuppMat.docx.

## Data Availability

Research data are not shared.
