# Supplementary material for: Graphene‐Skinned Alumina Fiber: Continuously Scalable Fabrication and Its Electrothermal Application in Fiber‐Reinforced Polymer Composites
Source: Adv Sci (Weinh). 2026 Mar 4;13(28):e24262. doi: 10.1002/advs.202524262 (PMC13185829; doi:10.1002/advs.202524262)
Supplement: Supplementary file 1 — Supporting File: advs74712‐sup‐0001‐SuppMat.docx. [file ADVS-13-e24262-s001.docx]

Supporting Information

Graphene-skinned alumina fiber: continuously scalable fabrication and its electrothermal application in fiber-reinforced polymer composites

*Xiaobai Wang*, Wenjing Jiang, Jingnan Wang, Yuejie Zhao, Enshan Liu, Fushun Liang, Yixin Zhang, Qingqing Liu, Kangyi Zheng, Yuyao Yang, Fan Yang, Xiao Jiang, Yue Qi*, Zhongfan Liu**

*E-mail: xiaobai_wang@yeah.net; zfliu@pku.edu.cn; qiyue@bgi-graphene.com.


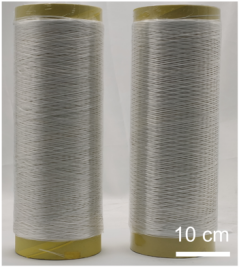


**Figure S1.** Photographs of commercial AF.


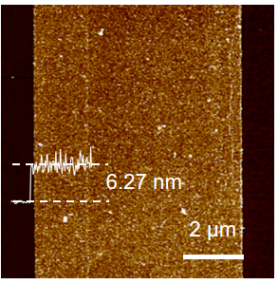


**Figure S2.** The atomic force microscopy (AFM) image of a graphene ribbon obtained by etching the core AF of GAF and the corresponding thickness value (~6.27 nm).

The core AF of GAF was etched with hydrofluoric acid, and the graphene layers were collapsed onto a silicon substrate, resulting in a bundle of graphene ribbons. The thickness measured by AFM was twice the actual thickness of the grown graphene layer plus 0.8 nm. The value of 0.8 serves as a correction that accounts for the increase in measured thickness, which arises from interactions for both the substrate-graphene and graphene-tip interactions, as well as the interlayer interactions within the graphene^[1]^.

Therefore, the corresponding thickness of the grown graphene layers was ~2.7 nm.


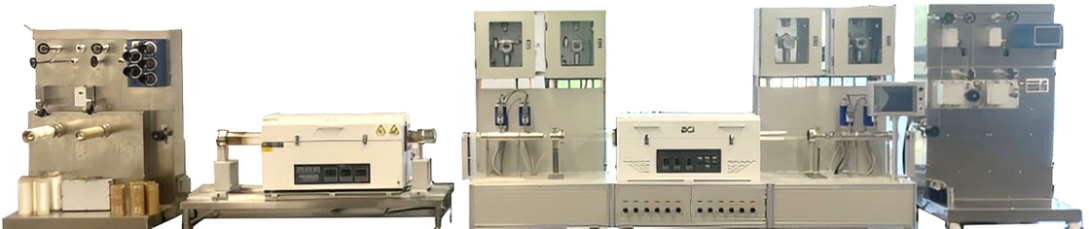


**Figure S3.** Photograph of the Dynamic, continuous, and scalable production system of GAF.


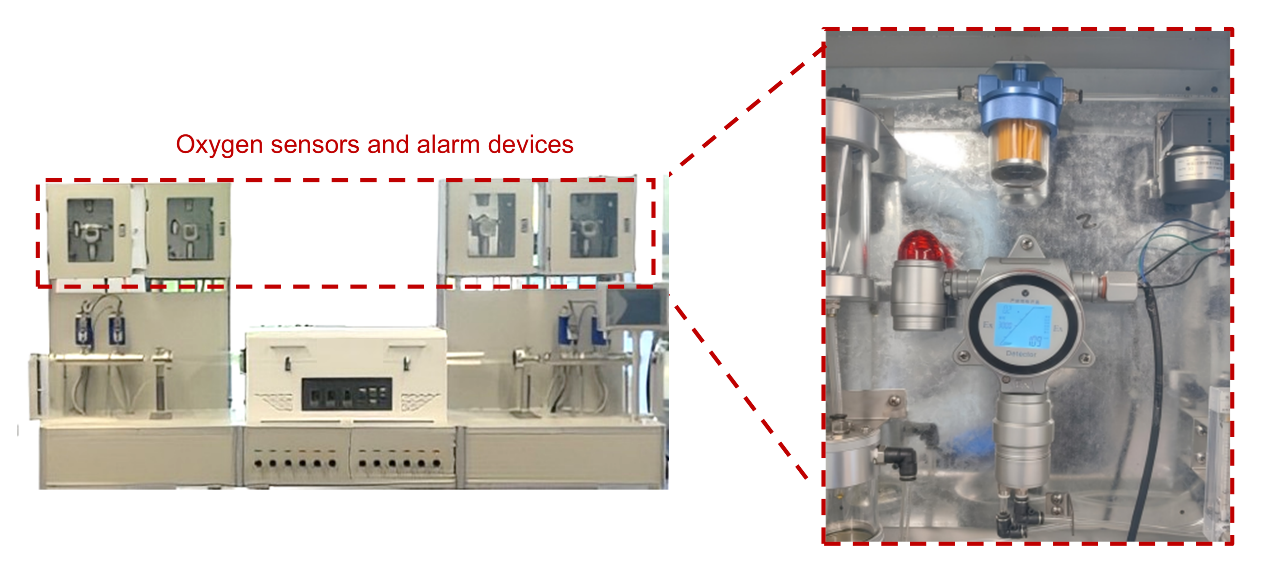


**Figure S4.** Photograph of the oxygen concentration detection device**,** and the corresponding oxygen concentration value (~1%).


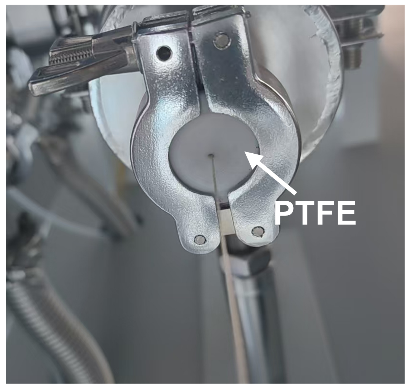


**Figure S5.** Photograph of a polytetrafluoroethylene (PTFE) gasket. PTFE serves as a physical barrier to prevent oxygen from penetrating the CVD chamber


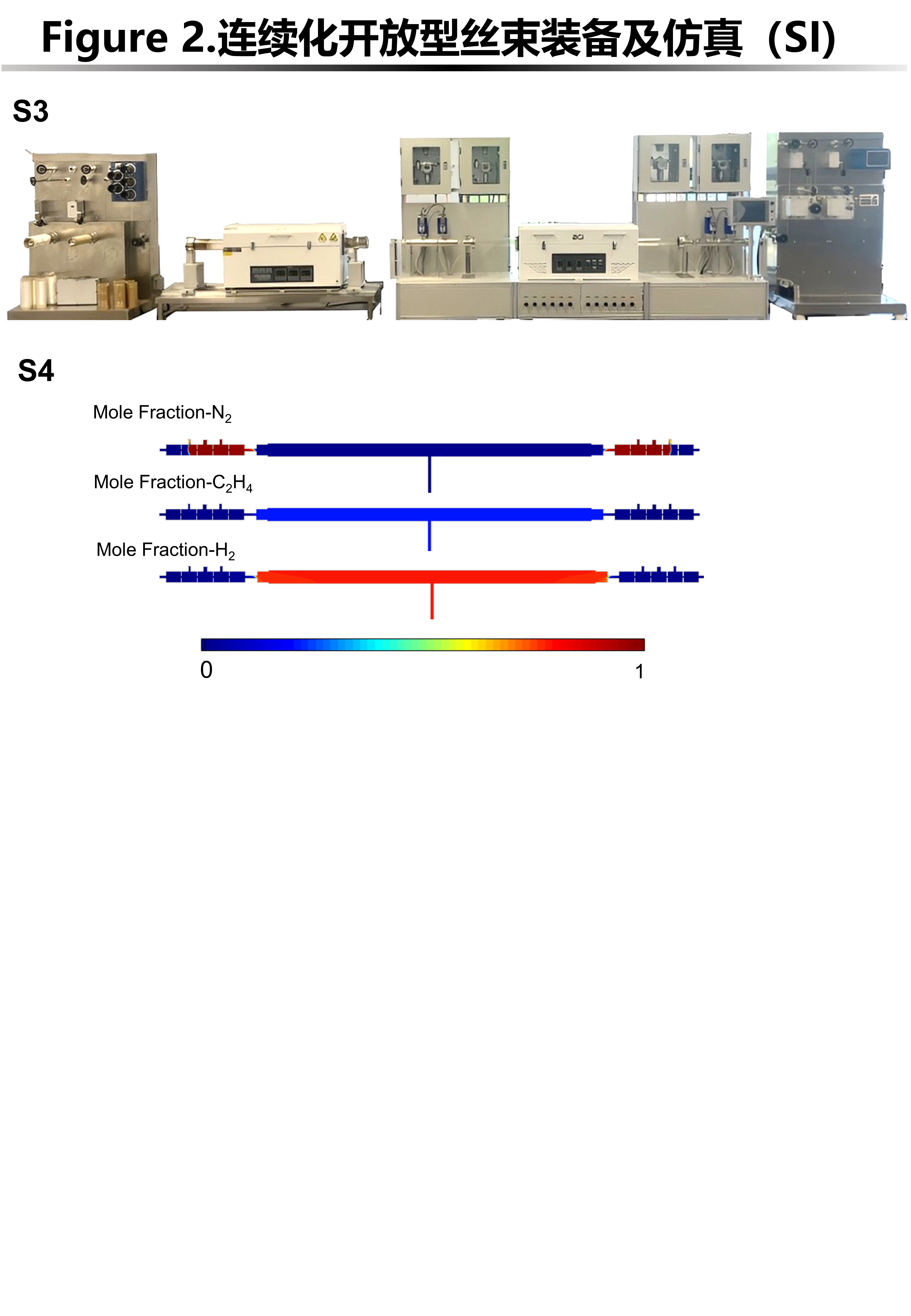


**Figure S6.** CFD simulation for the distribution of N_2_, C_2_H_4_ and H_2_ mole fraction.

**Note S1.** Detailed information on Computational Fluid Dynamics (CFD) simulation

**(1)** Boundary conditions

The boundary conditions were set based on the actual experiments. The inlet and outlet boundary conditions were set with boundaries of velocity, pressure and temperature. Specifically, the inlet boundary conditions for the N_2_ and mixed gas of C_2_H_4_ and H_2_ (mass fraction of C_2_H_4_: 0.2177) adopted the velocity-inlet boundary, with the inlet velocities being 0.62 m/s and 0.02 m/s, respectively. The air leakage inlet boundary condition adopted the mass-flow-inlet boundary, with the inlet mass flow rate being 0.00037 kg/s. All the outlets adopted the pressure-outlet boundary, with the outlet pressure being 0 Pa.

Through actual measurement, the pumps were capable of maintaining the chamber pressure inside the chamber at 9000 Pa. Therefore, the “operating pressure” of the “operating conditions” of the integrated device was set to 9000 Pa. Additionally, the “gravity” of the “operating conditions” was set to 9.8 m/s.

All wall boundaries of the integrated device adopted the temperature boundary. The wall temperature of the heating zone of the growth chamber is 1373.15 K, the wall temperatures at both ends of the growth chamber with liquid cooling devices are 293.15 K, and the wall temperature of the multi-stage pressure regulation device is 298.15 K.

**(2)** Solver settings

The Pressure-Velocity Coupling algorithm adopts SIMPLEC, with Skewness Correction set to 1. In the Spatial Discretization settings, Gradient was selected as Least Squares Cell Based, and Pressure, Momentum, Turbulent Kinetic Energy and Specific Dissipation Rate all adopted Second Order Upwind.

**(3)** Mesh partitioning

In the Integrated Computer Engineering and Manufacturing (ICEM) module of ANSYS software, the quadrilateral mesh partitioning method was adopted to mesh the geometric model. By densifying the mesh at the leakage outlet position, the calculation accuracy was improved. The total number of meshes was 720,000.


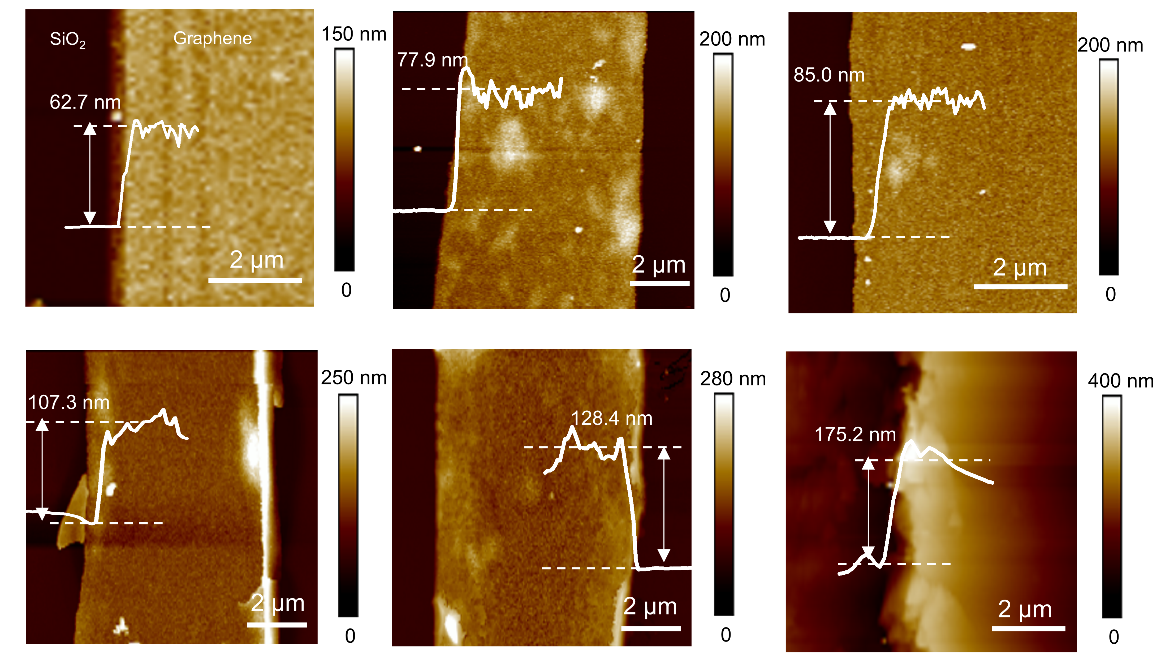


**Figure S7.** The AFM images of graphene ribbons obtained by etching the core AF of GAF and the corresponding thickness value of ~62.7 nm (a), ~77.9 nm (b), ~85.0 nm (c), ~107.3 nm (d), ~128.4 nm (e), ~175.2 nm (f). The corresponding thicknesses of the grown graphene layers were ~30.9, ~38.5, ~42.1, ~53.3, ~63.8, and ~87.2 nm, and the corresponding graphene layer numbers were ~92, ~115, ~126, ~159, ~191, and ~261, respectively. Growth conditions: ~1100 °C, rewinding/unwinding rate of 10 mm min^-1^ with C/H ratios of 1:25, 1:30, 1:35, 1:40, 1:45, 1:50.

**Table S1.** Statistics of graphene thickness, graphene layer number, and GAF conductivity at different C/H ratios

| C/H ratio | Thickness (nm) | Layer number | Conductivity (S m^-1^) | Count (n) |
| --- | --- | --- | --- | --- |
| 1:50 | 31.62 ± 2.13 | 95 | 674.08 ± 7.29 | 6 |
| 1:45 | 39.45 ± 2.40 | 118 | 757.24 ± 6.85 | 6 |
| 1:40 | 44.37 ± 2.69 | 134 | 796.66 ± 9.45 | 6 |
| 1:35 | 53.03 ± 3.27 | 159 | 896.28 ± 11.98 | 6 |
| 1:30 | 63.03 ± 3.18 | 188 | 1019.87 ± 12.30 | 6 |
| 1:25 | 84.22 ± 5.95 | 252 | 1238.19 ± 19.44 | 6 |


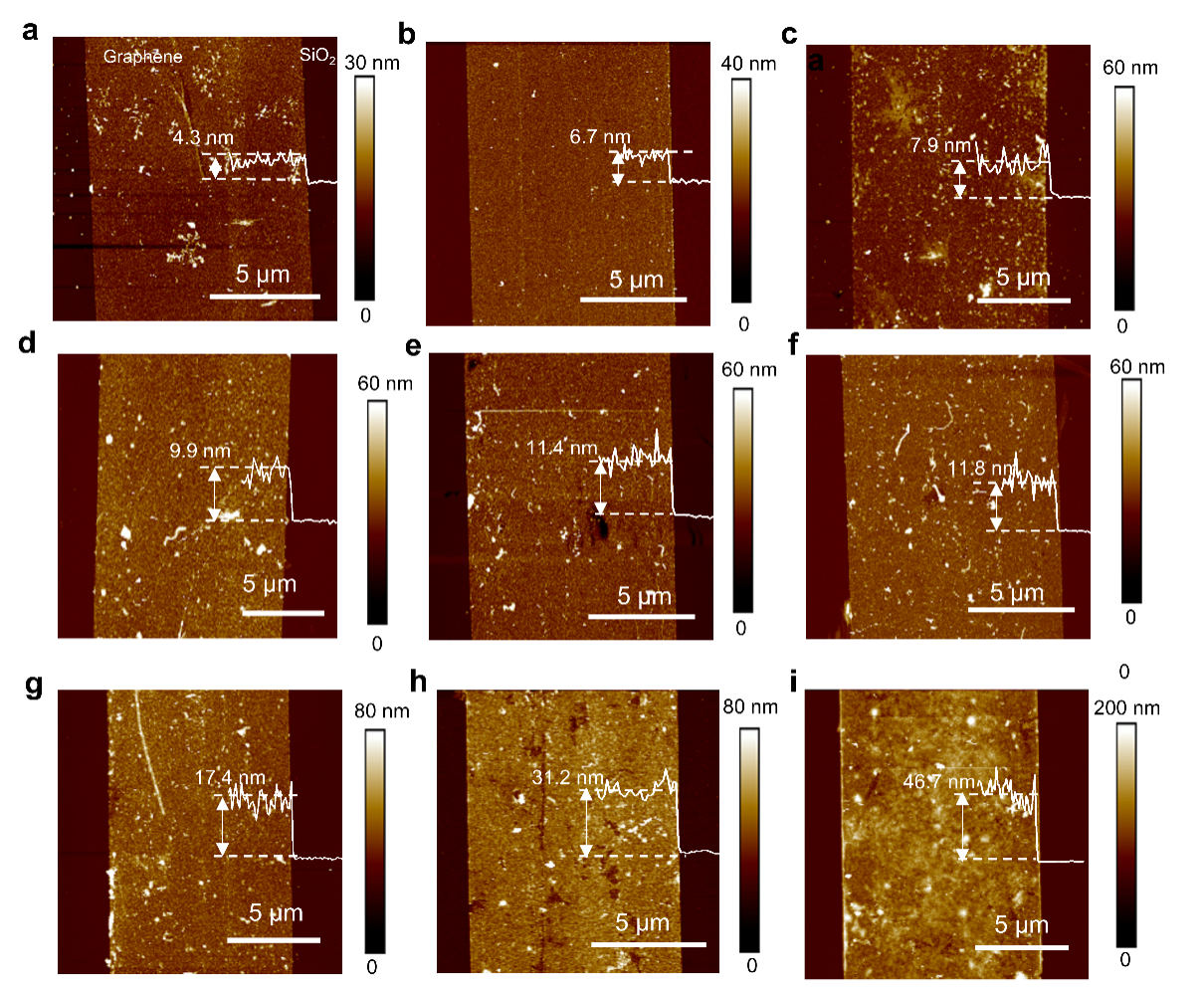


**Figure S8.** The AFM images of graphene ribbons obtained by etching the core AF of GAF and the corresponding thickness value of ~4.3 nm (a), ~6.7 nm (b), ~7.9 nm (c), ~9.9 nm (d), ~11.4 nm (e), ~11.8 nm (f), ~17.4 nm (g), ~31.2 nm (h), ~46.7 nm (i). The corresponding thicknesses of the grown graphene layers were ~1.8, ~3.0, ~3.6, ~4.6, ~5.3, ~5.5, ~8.3, ~15.2, ~23.0 nm and the corresponding graphene layer numbers were ~5, ~9, ~11, ~14, ~16, ~16, ~25 ~46 and ~69, respectively. Growth conditions: ~1100 °C, 10 sccm C_2_H_4_, 500 sccm H_2_ with rewinding/unwinding rates of 10-100 mm min^-1^.

**Table S2.** Statistics of graphene thickness, graphene layer number, and GAF conductivity at different rewinding/unwinding rates

| Rewinding/unwinding rate (mm min^-1^) | Thickness (nm) | Layer number | Conductivity (S m^-1^) | Count (n) |
| --- | --- | --- | --- | --- |
| 20 | 23.51 ± 1.74 | 70 | 274.79 ± 3.76 | 6 |
| 30 | 15.31 ± 0.94 | 46 | 170.19 ± 1.99 | 6 |
| 40 | 8.41 ± 0.61 | 25 | 125.56 ± 1.74 | 6 |
| 50 | 5.61 ± 0.42 | 17 | 90.20 ± 1.19 | 6 |
| 60 | 5.50 ± 0.39 | 16 | 78.66 ± 1.15 | 6 |
| 70 | 4.43 ± 0.30 | 13 | 64.36 ± 0.54 | 6 |
| 80 | 3.41 ± 0.24 | 10 | 58.56 ± 0.78 | 6 |
| 90 | 2.72 ± 0.14 | 8 | 51.56 ± 0.85 | 6 |
| 100 | 1.73 ± 0.17 | 5 | 43.40 ± 0.86 | 6 |


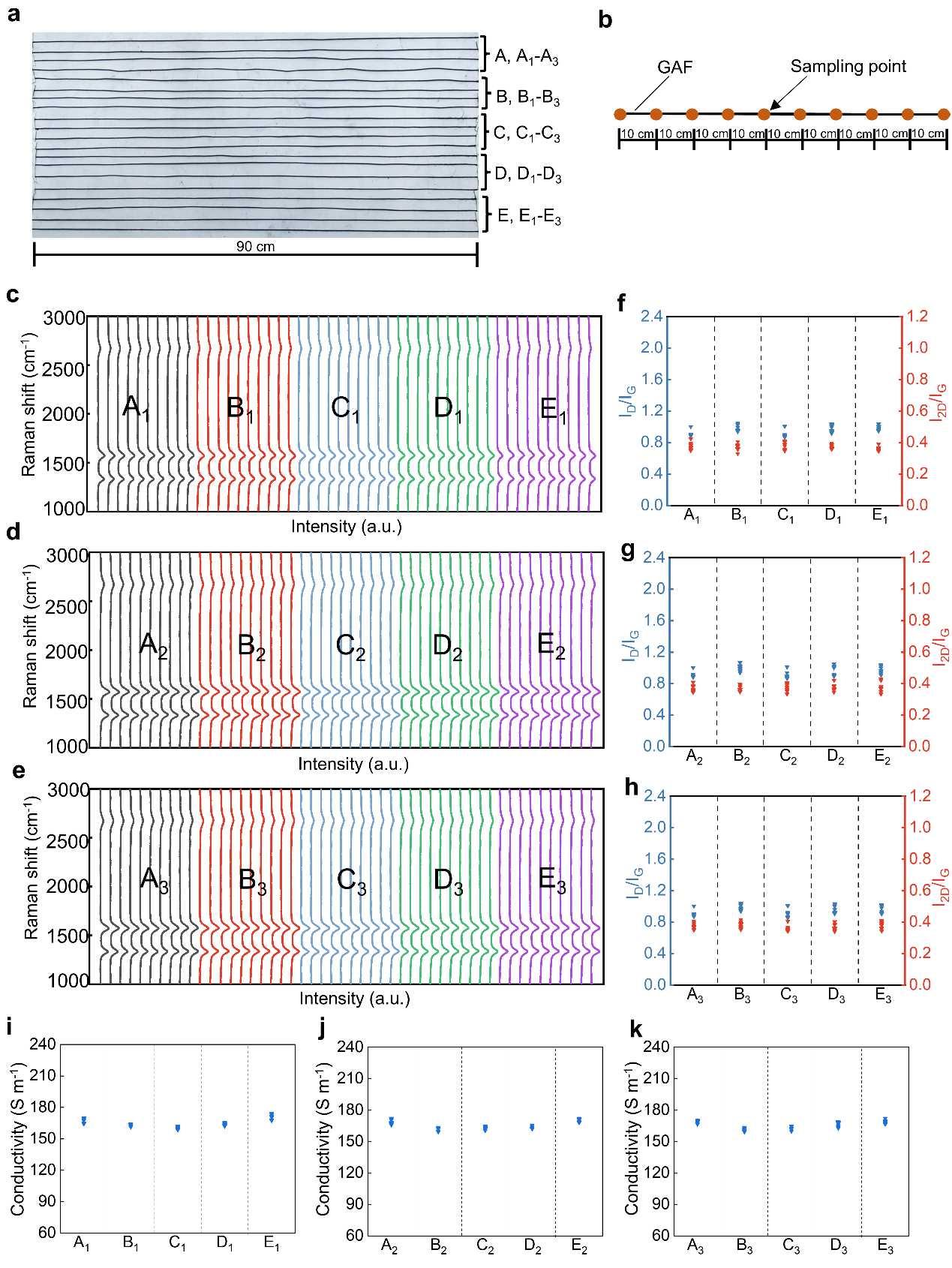


**Figure S9**. **a**) Photograph of twenty GAFs (with a length of ~90 cm) obtained from five different batches (A-E, A_1_-E_1_, A_2_-E_2_, A_3_-E_3_), each under identical growth conditions. **b**) Schematic illustrating the collection positions of Raman spectra and conductivity, with measurement points evenly spaced at ~10 cm intervals along the central axis. **c-e**) Raman spectra collected from five different batches of GAF (A_1_-E_1_, A_2_-E_2_, A_3_-E_3_) at the labeled positions in (**b**). **f-h**) Raman intensity ratios *I_D_/I_G_* and *I_2D_/I_G_* derived from the Raman spectra in (**c-e**). **i-k**) Conductivity collected from five different batches of GAF (A_1_-E_1_, A_2_-E_2_, A_3_-E_3_) at the labeled positions in (**b**).

To evaluate the inter-batch stability of GAF, a stratified sampling strategy was employed. Five independently prepared batches were investigated (Total length per batch: 10 m; Growth conditions: 10 sccm C_2_H_4_, 500 sccm H_2_, growth temperature of ~1100 °C, and rewinding/unwinding rate of ~30 mm min^-1^). For each batch, four samples were randomly collected, each with a length of 90 cm. **Figure S9a** presents twenty GAFs obtained from the five different batches (A-E, A_1_-E_1_, A_2_-E_2_, A_3_-E_3_). All GAFs showed a uniform appearance. The Raman spectra of graphene and the conductivity of GAF were acquired for these samples. As shown in **Figure S9b**, for the Raman characterization of graphene in the GAF, spectra were collected at ten equally spaced positions along a transverse distance of ~90 cm on the central axis of GAF from the 5 batches in **Figure S9a**, with a uniform spacing of ~10 cm. The Raman spectra obtained in **Figures 3e and S9c-e** show almost identical D and G peak signals between different batches and within the same batch. The intensity ratio of D and G peaks (*I_D_*/*I_G_*) and the intensity ratio of 2D and G peaks (*I_2D_*/*I_G_*) are common metrics to evaluate graphene crystallinity and layer thickness, respectively. As shown in **Figures 3f and S9f-h**. The calculated *I_D_*/*I_G_* and *I_2D_*/*I_G_* values in the Raman spectra of the five batches of GAF (A-E, A_1_-E_1_, A_2_-E_2_, A_3_-E_3_) show approximately the same values, revealing consistent crystalline quality and layer thickness of graphene in the GAF both inter- batch and intra- batch.

For the conductivity measurements of GAF, conductivities were collected between two adjacent sampling points in **Figure S9b**, yielding a total of nine evenly spaced intervals, with uniform intervals of ~10 cm over a lateral distance of ~90 cm along the central axis of GAF from the 5 batches in **Figure S9a.** As shown in **Figures 3g and S9i-k**, the conductivity measurements across the five batches of GAF (A-E, A_1_-E_1_, A_2_-E_2_, A_3_-E_3_) exhibit excellent consistency and stability both inter- batch and intra- batch.

Based on the Raman intensity ratios *I_D_/I_G_* and *I_2D_/I_G_* derived from the Raman spectra and conductivity data obtained from the aforementioned sampling described above, the corresponding statistical results in **Figures 3f-g** and **S9f-k** are summarized in **Table S3**. The statistics show that the *I_D_*/*I_G_*, *I_2D_/I_G_* and conductivity are highly uniform and consistent across the five different batches (A-E, A_1_-E_1_, A_2_-E_2_, A_3_-E_3_), further confirming the inter-batch and intra-batch stability of GAF of the scaled-up production.

**Table S3**. Statistics of *I_D_*/*I_G_*, *I_2D_/I_G_* in Raman spectra and conductivity in Figures 3f-g in the main text and Figures S9f-k.

| Batch | *I*_2D_/*I*_G_ | *I*_D_/*I*_G_ | Count (n) | Conductivity (S m^-1^) | Count (n) |
| --- | --- | --- | --- | --- | --- |
| A | 0.37 ± 0.01 | 0.93 ± 0.07 | 10 | 166.72 ± 1.09 | 9 |
| A_1_ | 0.37 ± 0.02 | 0.93 ± 0.05 | 10 | 167.32 ± 1.98 | 9 |
| A_2_ | 0.37 ± 0.02 | 0.92 ± 0.04 | 10 | 168.56 ± 2.18 | 9 |
| A_3_ | 0.38 ± 0.02 | 0.92 ± 0.05 | 10 | 169.17 ± 1.30 | 9 |
| B | 0.38 ± 0.01 | 0.99 ± 0.06 | 10 | 163.87 ± 0.76 | 9 |
| B_1_ | 0.37 ± 0.02 | 0.99 ± 0.04 | 10 | 163.04 ± 1.11 | 9 |
| B_2_ | 0.37 ± 0.02 | 0.99 ± 0.04 | 10 | 161.72 ± 1.59 | 9 |
| B_3_ | 0.38 ± 0.02 | 0.99 ± 0.04 | 10 | 161.40 ± 1.57 | 9 |
| C | 0.37 ± 0.02 | 0.93 ± 0.05 | 10 | 162.36 ± 0.95 | 9 |
| C_1_ | 0.38 ± 0.02 | 0.92 ± 0.06 | 10 | 160.57 ± 1.25 | 9 |
| C_2_ | 0.37 ± 0.02 | 0.93 ± 0.06 | 10 | 162.96 ± 1.52 | 9 |
| C_3_ | 0.37 ± 0.02 | 0.93 ± 0.06 | 10 | 162.30 ± 1.64 | 9 |
| D | 0.38 ± 0.01 | 0.97 ± 0.03 | 10 | 165.40 ± 0.64 | 9 |
| D_1_ | 0.37 ± 0.01 | 0.97 ± 0.04 | 10 | 163.79 ± 1.29 | 9 |
| D_2_ | 0.37 ± 0.02 | 0.97 ± 0.06 | 10 | 163.62 ± 1.10 | 9 |
| D_3_ | 0.37 ± 0.02 | 0.97 ± 0.05 | 10 | 166.02 ± 2.03 | 9 |
| E | 0.38 ± 0.01 | 0.98 ± 0.07 | 10 | 168.37 ± 1.12 | 9 |
| E_1_ | 0.36 ± 0.01 | 0.99 ± 0.03 | 10 | 171.00 ± 2.09 | 9 |
| E_2_ | 0.38 ± 0.03 | 0.98 ± 0.05 | 10 | 170.16 ± 1.34 | 9 |
| E_3_ | 0.38 ± 0.02 | 0.98 ± 0.04 | 10 | 168.46 ± 1.66 | 9 |

**Note S2.** Calculation for the annual production capacity of GAF

The annual production of GAF is related to the conductivity of the required GAF and the preparation process. For example, to obtain GAF with a conductivity of ~1200 S m^-1^, the preparation conditions were C_2_H_4_ 10 sccm, H_2_ 250 sccm, the rewinding and unwinding rate was 10 mm min^-1^. One roll of AF is ~300 m in length. By considering the equipment having 10 days downtime over a year (comprising 3 days for scheduled maintenance/servicing and roll changing; 1 day for cleaning the chamber heating zone prone to carbon deposition/contamination and contaminated gas path; 2 day for pressure-holding maintenance of multi-stage pressure regulation modules and temperature calibration; 1 day for tuning and calibrating the rewinding and unwinding rate; 2 days for precursor gas replacement or gas-path calibration; and 1 day for unscheduled downtime (e.g. failures or gas leakage). Therefore, the effective annual operating time is calculated as 355 days.

The annual production of GAF is calculated as follows:

$$\left( 355\times24 \right) h\times\left( 10\times60\times\frac{1}{1000} \right) m/h\approx5100 m$$

Similarly, to obtain GAF with a conductivity of 40 S m^-1^, the preparation conditions were C_2_H_4_ 10 sccm, H_2_ 250 sccm, the rewinding and unwinding rate was 300 mm min^-1^. One roll of AF is ~300 m in length, and the effective annual operating time is calculated as 355 days.

The annual production of GAF is calculated as follows:

$$\left( 355\times24 \right) h\times\left( 300\times60\times\frac{1}{1000} \right) m/h\approx153000 m$$

In summary, depending on the GAF specifications, the annual production capacity is in the range of 5100-153000 m.


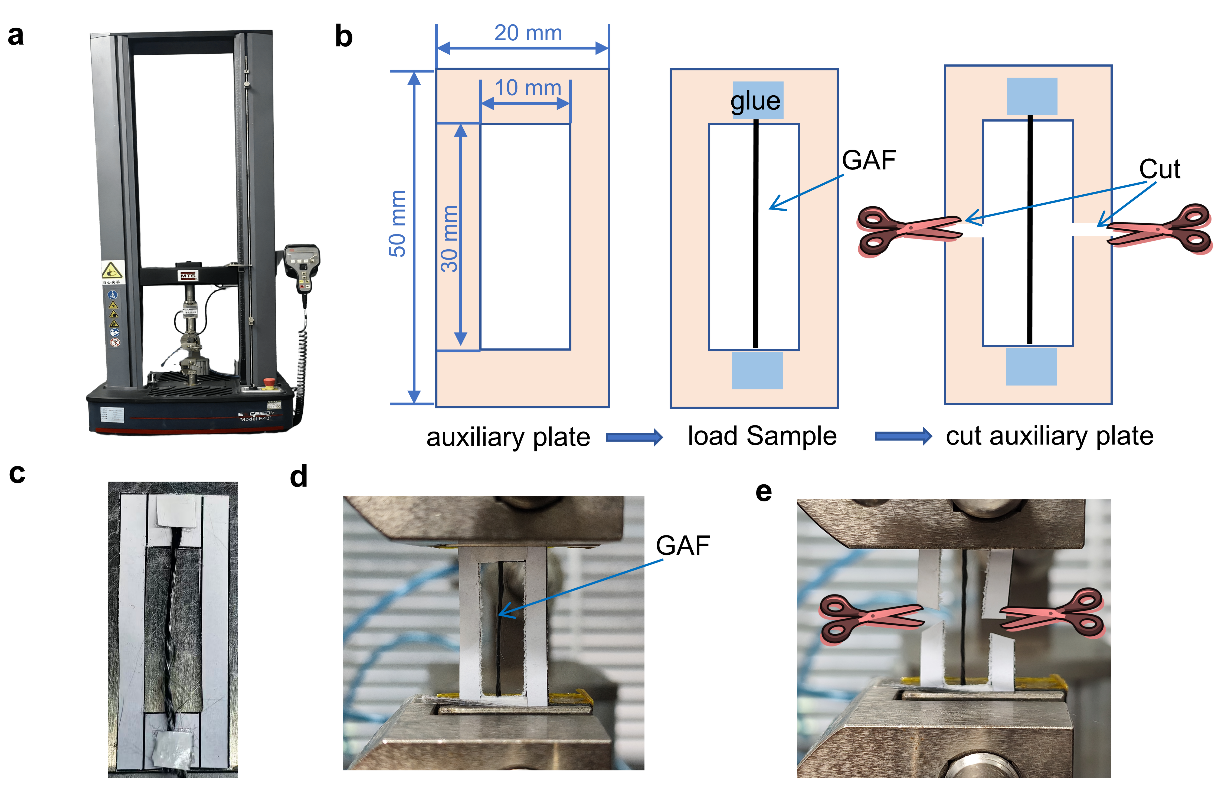


**Figure S10.** The tensile strength measurement process of GAF. **a**) The photograph of the electronic universal material testing machine (MTS E43.104) used for tensile strength measurement of GAF. **b, c**) Schematic diagrams (b) and a photograph (c) of the GAF being loaded on the auxiliary plate before the test. **d, e**) A photograph of the GAF loaded on the auxiliary board being fixed in the upper and lower fixtures of the machine


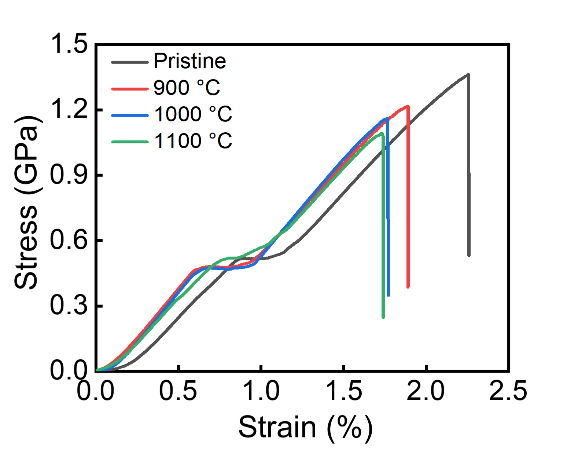


**Figure S11.** Tensile stress-strain curves of pristine AF and GAF after graphene CVD growth at different temperatures (~900 °C, ~1000 °C, ~1100 °C for 10 sccm C_2_H_4_, 500 sccm H_2_ and rewinding/unwinding rate of ~10 mm min^-1^).


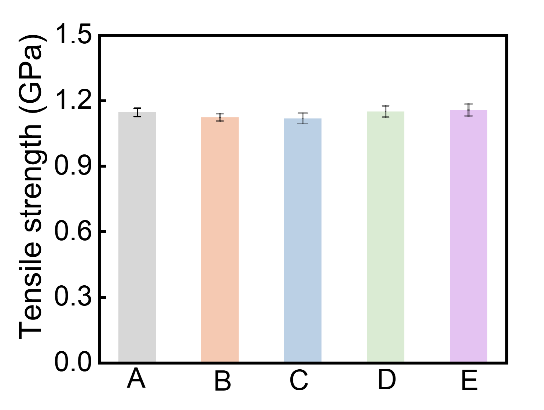


**Figure S12.** Tensile strength of GAFs from the 5 batches (A-E). Error bars represent the standard deviations (n=5).


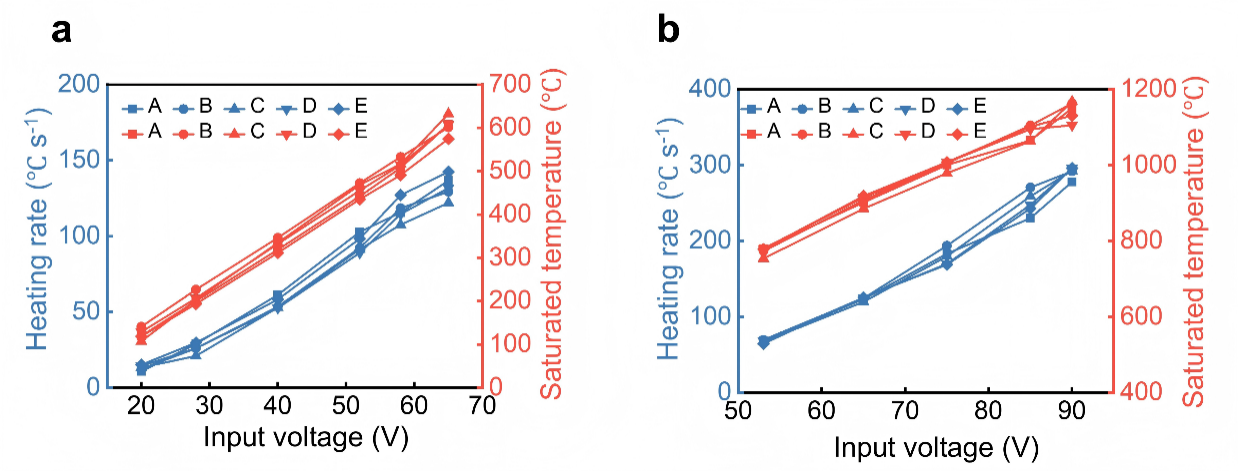


**Figure S13.** Heating rates and saturated temperatures of GAFs from the 5 batches (A-E) under different input voltages in an air (a) and vacuum environments (b). The size of GAFs is all 10 cm. Growth conditions: 10 sccm C_2_H_4_, 500 sccm H_2_, growth temperature of ~1100 °C, and rewinding/unwinding rate of ~10 mm min^-1^.


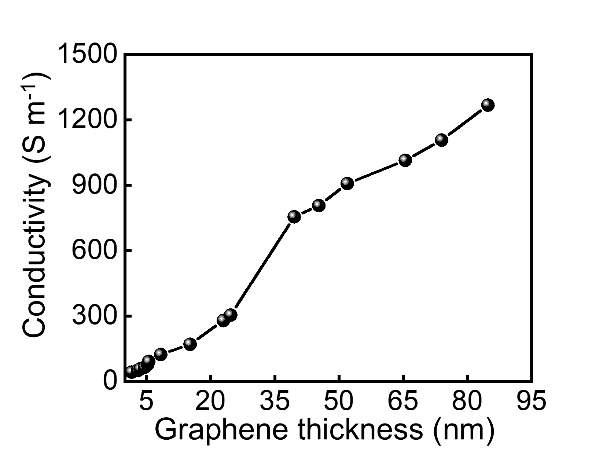


**Figure S14.** Effect of graphene thickness of GAF heating wire on the conductivity of GAF.


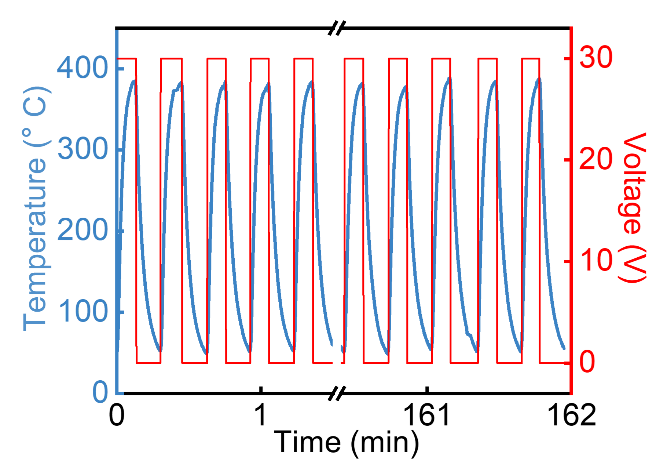


**Figure S15.** Temperature response of GAF heating wire under square wave voltage cycles in an air environment (0-30 V, a period of 16 s, ~607 continuous cycles).


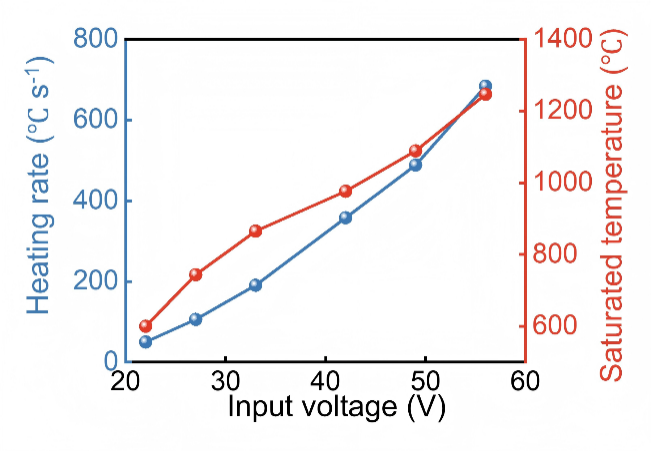


**Figure S16.** Heating rate and saturated temperature of GAF heating wire under different input voltages in a vacuum environment.


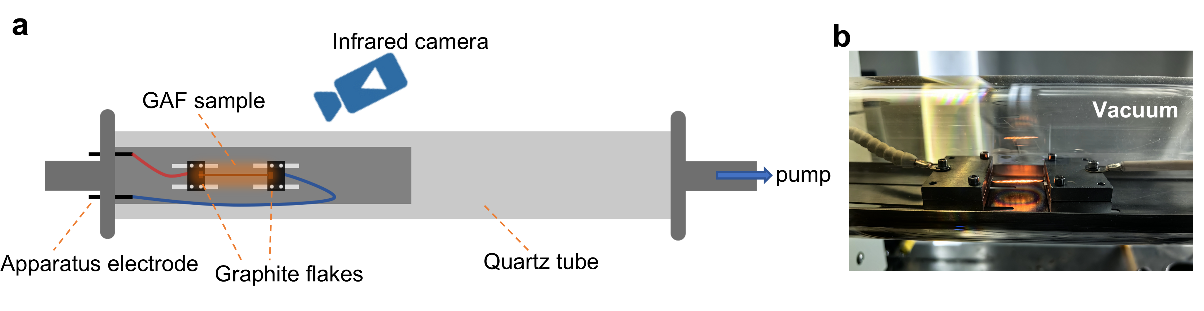


**Figure S17.** a, b) Schematic diagram (a) and photograph (b) for the high-temperature heating test apparatus under vacuum of GAF.

We developed the heating apparatus for GAF, as shown in **Figure S17a**. This heating apparatus consists of a circular baffle, a flat guide rail, and two movable graphite flakes. The entire apparatus is placed in a quartz tube. The circular baffle is positioned at the opening of the quartz tube on the left to seal it. The electrodes on the two graphite flakes are integrated onto the circular baffle to establish an electrical connection to the external power supply, while the quartz tube without breaking its sealing. The upper and lower graphite flakes are arranged to clamp the GAF sample, and the length and position of the sample can be adjusted according to the flat guide rail.

During the experiment, the quartz tube was pumped to a vacuum (~ 10 Pa) by a vacuum pump. Then, 10 sccm of H_2_ as the reducing gas and 100 sccm of Ar as inert gas were introduced. The direct current (DC) power supply was connected to the integrated electrodes on the circular baffle. After applying voltage to the electrodes at both ends of GAF, the infrared images of GAF were captured in real time using an infrared camera (Fluke Ti 10). Both the heating and cooling curves were recorded to assess the dynamic thermal response of the GAF. The photograph of the high-temperature vacuum heating of GAF is shown in **Figure S17b**, and GAF presents a uniform appearance during heating.


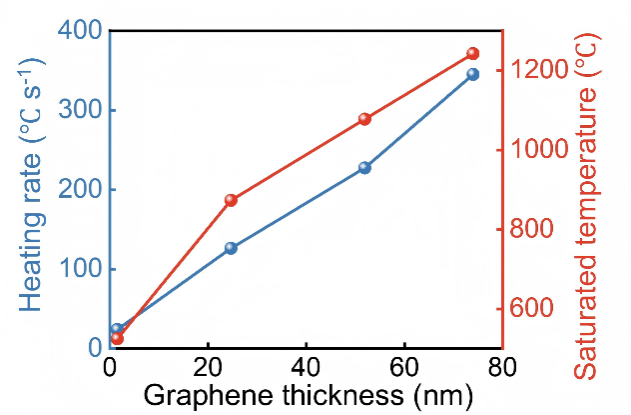


**Figure S18.** Effect of graphene thickness of GAF heating wire on heating rate and saturated temperature in a vacuum environment (The size of GAFs is all 10 cm, the input voltage is all ~80 V).


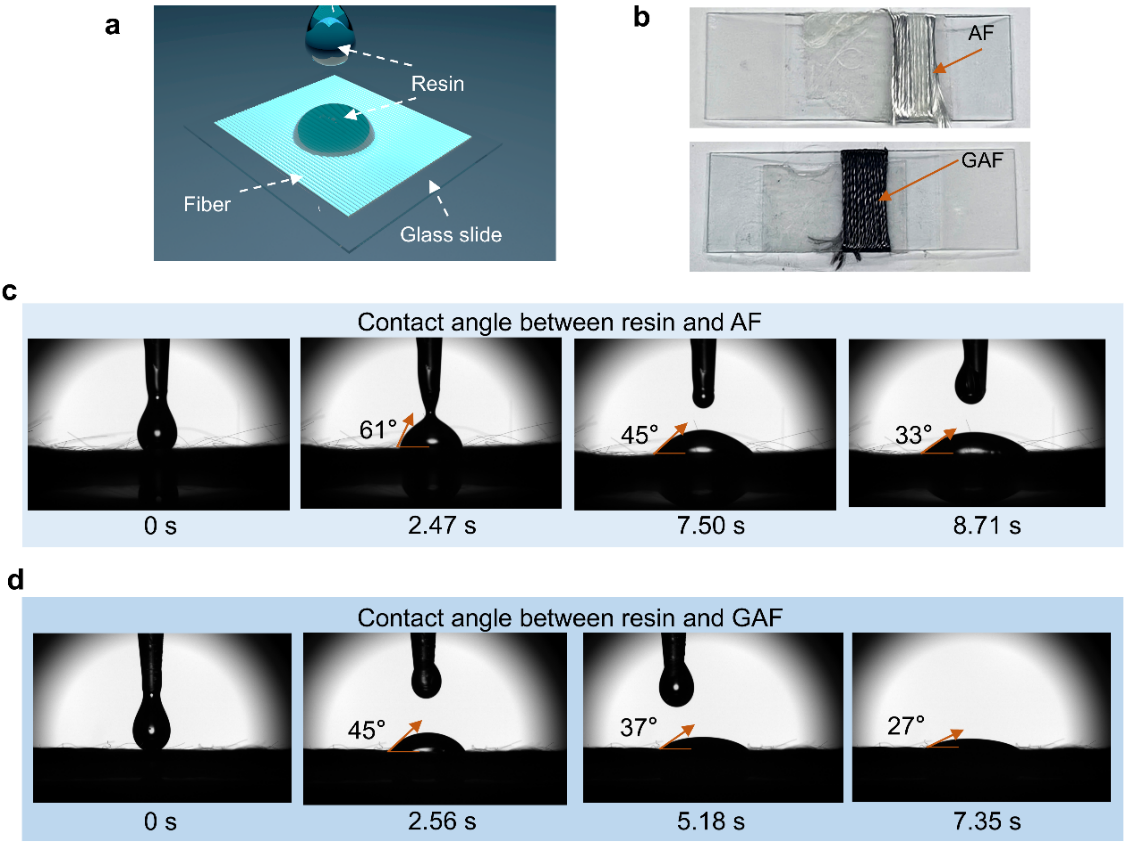


**Figure S19.** a) Schematic of dynamic contact angle measurement of resin on unidirectionally arranged fibers via droplet-profile optical analysis. b) Photograph of unidirectionally arranged AFs and GAFs on the slide. c, d) Dynamic contact angle measurement of resin on unidirectionally arranged AFs and GAFs.

In view of the actual processing conditions of GAF and AF during fiber-reinforced polymer (FRP) composites manufacturing, we record the spreading of epoxy droplets on unidirectionally arranged GAFs or AFs. As shown in Figures S19a and S19b, the AFs and GAFs were wrapped on the slide in a unidirectional arrangement, and the slide was placed flatly on the sample observation stage. The epoxy droplets were slowly dripped onto the surface of the arranged fibers from a syringe above, and the spreading process of the epoxy droplets on the fiber was recorded by a high-speed camera.

Figure S19c and Figure S19d show the change of droplet shape and contact angle with time during the spreading process of epoxy droplets on the surface of unidirectionally arranged AFs and GAFs. It can be seen that the contact angle of the epoxy droplet on the unidirectionally arranged AFs’ surface decreased to 33° after 8.71 s. However, the contact angle of epoxy droplets on the unidirectional GAFs’ surface decreased to 27° after 7.35 s, and the resin showed better spreading and wetting ability.


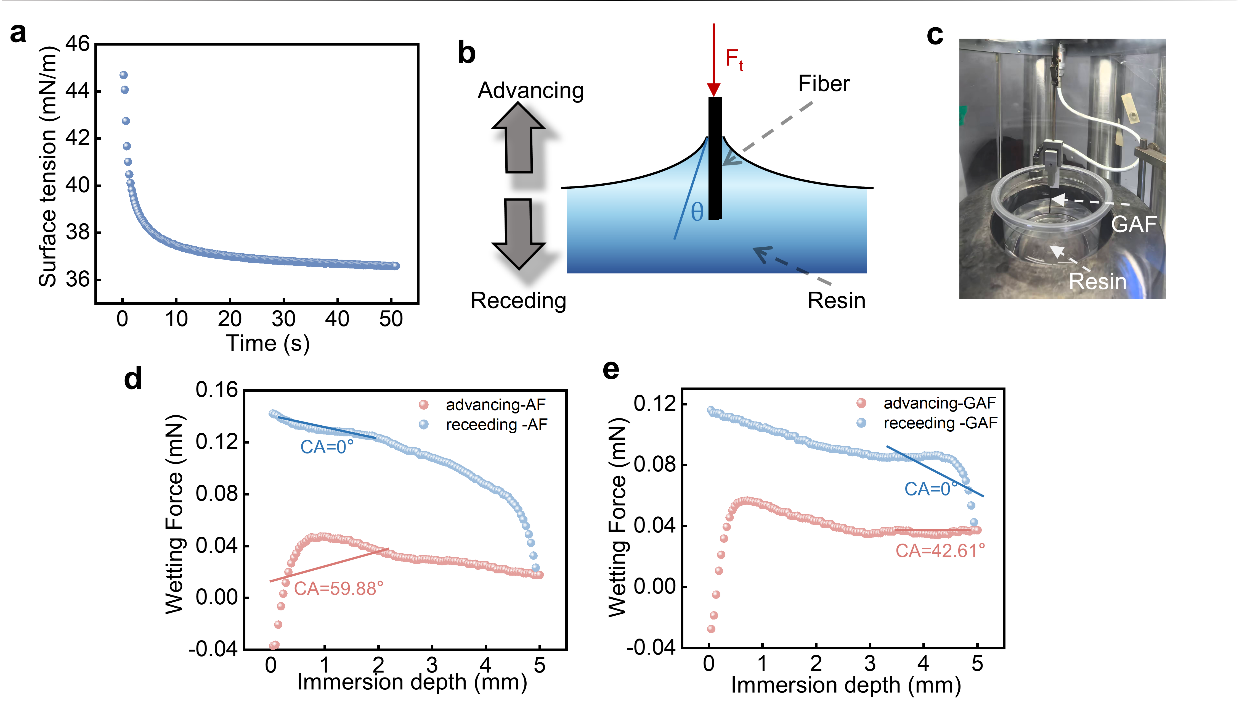


**Figure S20.** a) Dynamic surface tension of the resin as a function of time. b) Schematic of dynamic contact angle measurement between the resin surface and the fiber via the Wilhelmy method. c) Photograph of dynamic contact angle measurement between the resin surface and the fiber via the Wilhelmy method. d, e) The variation of the wetting force on the AF and GAF with the immersion depth (The advancing angle and receding angle are obtained when the wetting force reaches equilibrium).

The surface tension value of epoxy resin (G6021A) can be measured by using a surface tension measuring instrument. Figure S20a shows the change of dynamic surface tension of the resin. The dynamic surface tension $\gamma(t)$ of the resin was recorded from the moment of liquid-gas interface formation (t=0). 𝛾 (𝑡) gradually decreases with time and enters a quasi-steady state after ~44 s, from which the equilibrium surface tension is obtained to be 36.63 mN/m.

As shown in Figure S20b, the dynamic contact angles (advancing and receding angles) between the fiber and the epoxy resin were measured by the Wilhelmy method. When the fiber is immersed in the resin, it is subjected to a force $F_{t}$ exerted by the surface tension of the resin, which can be determined by weighing.

Figure S20c shows the photograph of the dynamic contact angle measurement (advancing and receding angles) between the fiber and the epoxy resin. During the measurement, a single AF or GAF was fixed vertically on the microbalance probe, and immersed (advanced) and withdrawn (receded) from the resin at a constant speed, with the wetting force $F_{t}$ recorded in real time as a function of immersion depth. The advancing and receding angles can be calculated by the Wilhelmy equation:

$$F_{t}=p\gamma_{LV}cos\theta+mg-\rho gV-F_{0}$$

Where $F_{t}$ is the wetting force, $p$ is the wetted perimeter of the fiber ($p=\pi d$, where $d$ is the fiber diameter), $\gamma_{LV}$ is the surface tension of the resin, $\theta$ is the contact angle, $m$ is the mass of the fiber, $g$ is the gravitational acceleration, $\rho$ is the density of the resin, $V$ is the immersed volume of the fiber ($V=Az$, where $A$ is the cross-sectional area of the fiber and $z$ is the immersion depth), $F_{0}$ is baseline-corrected force, with the fiber weight automatically zeroed before liquid immersion.

Figure S20d shows the variation of the wetting force on the AF with the immersion depth. The wetting force on the AF increases with immersion depth (rising resin liquid level), and then plateaus, corresponding to the advancing angle reaching an equilibrium value of ~60°. When the fiber is withdrawn from the resin, the equilibrium wetting force corresponds to a receding angle of 0°, indicating strong adhesive and wetting forces between the resin and the AF surface, resulting in the resin not readily withdrawing from the AF surface. Correspondingly, Figure S20e shows the variation of the wetting force on the GAF with the immersion depth. Resin and GAF showed similar infiltration behavior to AF. When the wetting force on GAF tended to be balanced, the corresponding equilibrium advancing angle was 42.61°, indicating that the infiltration ability of resin on GAF was stronger than that of AF. During the withdrawal stage, the receding angle was 0°, indicating that the resin maintains excellent wettability and strong adhesion to the GAF.


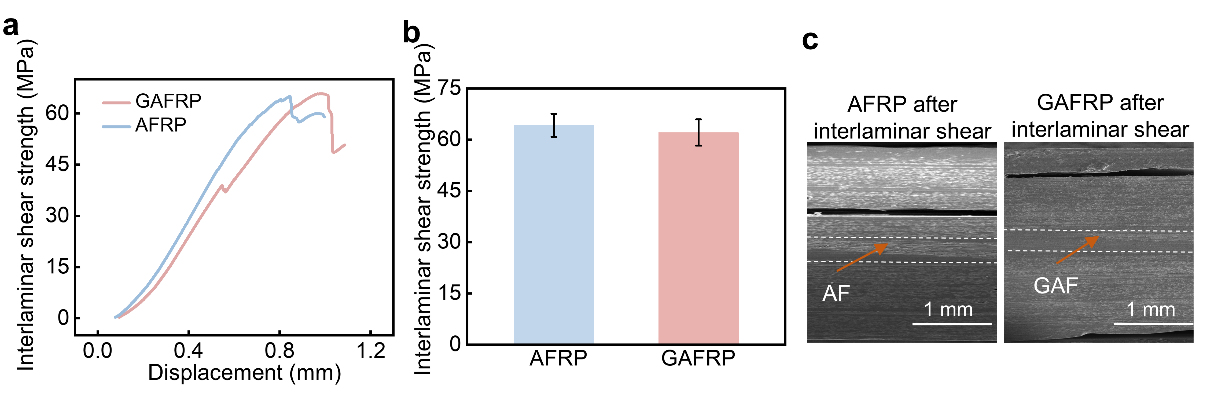


**Figure S21.** a) Interlaminar shear strength of AFRP and GAFRP composite as a function of displacement. b) Maximum interlaminar shear strength of AFRP and GAFRP composite. The error bars represent the standard deviations (n=4). c) SEM cross-sectional morphology of AFRP and GAFRP composite after interlaminar shear failure.


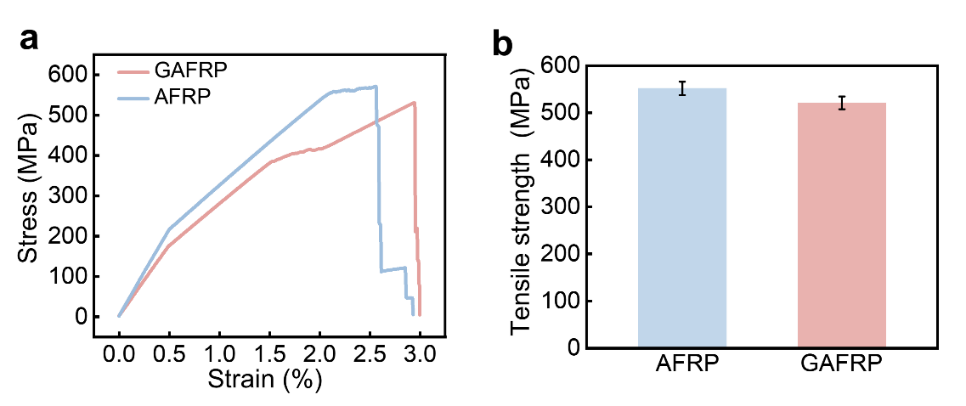


**Figure S22.** a) Tensile stress-strain curves of AFRP and GAFRP (wherein GAFs were prepared at a growth temperature of 1100 °C) b) Tensile strength of AFRP and GAFRP. The error bars represent the standard deviations (n=3).

For the AFRP and GAFRP, 10-layer glass fiber unidirectional prepregs were layup, while the parallel and non-interwoven GAFs or AFs are placed in the middle layer (fiber laying density: 0.5 per cm^2^; fiber spacing: 2 mm), wherein the GAFs were prepared at a growth temperature of 1100 ℃. Subsequent thermal curing produced the final AFRP and GAFRP composite. The composite specimens were cut to dimensions specified by ASTM D2344 (250 mm ×15 mm ×1 mm) and subjected to tensile strength measurement.


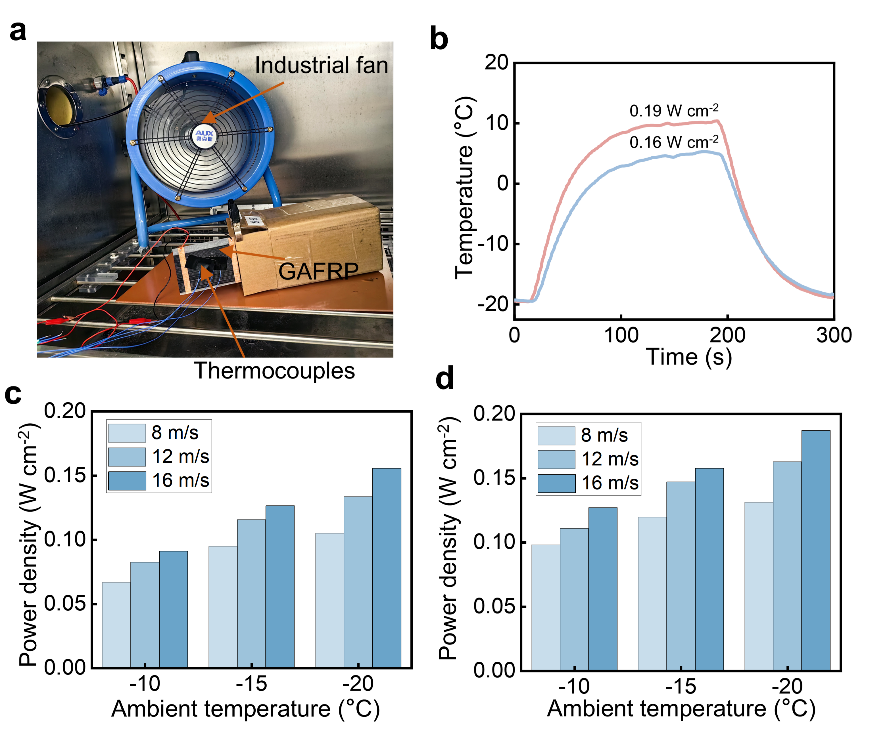


**Figure S23.** a) Photograph of the GAFRP's electrothermal test in a convective cooling environment. b) Temperature profiles of the GAFRP heating plate under different input power densities (wind speed:16 m/s; ambient temperature: -20 °C). c, d) Steady-state power densities required to achieve GAFRP’s surface temperatures of 5 °C and 10 °C under different wind speeds and ambient temperatures.

As shown in Figure S23a, an industrial fan was placed in the freezer chamber to simulate a convection cooling environment. The wind speed was adjusted to 8 m/s, 12 m/s and 16 m/s, and ambient temperatures were set at -10 °C, -15 °C and -20 °C.

The sample is a GAF-reinforced polymer (GAFRP) composite. Multi-layer glass fiber unidirectional prepregs of different orientations (0°/90°/0°/+45°/-45°) were layup, while the parallel and non-interwoven GAFs are placed in the middle layer (bundle laying density: 0.5 per cm^2^; bundle spacing: 2 mm), and copper electrodes were affixed on both sides of the GAF bundles. Subsequent thermal curing produced the final GAFRP composite. The overall size is 120 mm × 100 mm × 10.4 mm. Drill holes at both ends of GAFRP to connect copper wires to the electrodes. Four K-type thermocouples were laid on the sample surface to monitor the surface temperature of the sample in real time.

A direct current power supply was used to apply voltage to the copper wires at both ends of GAFRP, and the data acquisition system recorded the temperature data from all thermocouples in real time. Whenever the ambient temperature or wind speed changes, the input power is adjusted to ensure that the surface temperature of the sample remains at 5 °C or 10 °C. Corresponding input power and heating/cooling curves were recorded under each condition. The power density of GAFRP can be calculated by the following formula:

$$P_{d}=\frac{P}{A}$$

Where $P_{d}$ is power density (W/cm^2^), P is input power (W), and $A$ is effective heating area (cm).

As shown in Figure S23b, the heating/cooling curves of GAFRP under different power density is presented (wind speed:16 m/s; ambient temperature: -20 °C). When the heat generated by Joule heating is balanced with heat dissipation, the GAFRP reached the corresponding saturated temperatures (5 °C and 10 °C), indicating an adjustable heating temperature range.

As shown in Figures S23c and S23d, the steady-state power densities required to reach GAFRP's surface temperatures of 5 °C and 10 °C under different wind speeds and ambient temperatures are demonstrated. When the wind speed is 16 m/s and the ambient temperature is -20 °C, to maintain the GAFRP surface temperature at 5 °C, only a power density of 0.16 W cm^-2^ is required, demonstrating the high electrothermal conversion efficiency of GAFRP. Additionally, under otherwise identical conditions, higher surface temperature. or increasing wind speeds, or lower ambient temperature demand higher input power or power density to demand thermal equilibrium.

**Note S3.** Detailed growth conditions of GAF

(1) Growth conditions in **Figures 1b-g**: 10 sccm C_2_H_4_, 500 sccm H_2_, growth temperature of ~1100 °C, rewinding and unwinding rate of ~50 mm min^-1^.

(2) Growth conditions of GAF under different C/H ratios (1:25, 1:30, 1:35, 1:40, 1:45, 1:50): 10 sccm C_2_H_4_, growth temperature of ~1100 °C, rewinding/unwinding rate of 10 mm min^-1^

(3) Growth conditions of GAF under different rewinding/unwinding rates (10-100 mm min^-1^): 10 sccm C_2_H_4_, 500 sccm H_2_, growth temperature of ~1100 °C

(4) Growth conditions of five different batches of GAF: 10 sccm C_2_H_4_, 500 sccm H_2_, growth temperature of ~1100 °C, rewinding and unwinding rate of ~30 mm min^-1^.

**Note S4.** Calculation of GAF conductivity based on the total cross-sectional area of the graphene layers and AF

$$\text{G=}\frac{\text{1}}{\text{ρ}}\text{=}\frac{\text{L}}{\text{RS}}=\frac{\text{L}}{\text{R×[}\text{π}\text{(r+t)}^{2}]} \left( 1 \right)$$

For example, for GAF with a graphene thickness of 1.7 nm, the measured length is 5 cm, the measured bulk resistance of GAF is 8370 Ω, and the radius of AF is 0.212 mm. Through equation (1), the conductivity of GAF can be calculated:

$$\text{G=}=\frac{\text{L}}{\text{R×[π}\text{(r+t)}^{2}]}$$

$$= \frac{\text{5×}{10}^{-2}}{\text{8370×[π×}{\text{(0.212×}{10}^{-3}\text{+1.7×}{10}^{-9}\text{)}}^{2}]}\approx42 (S\cdot m^{-1})$$

For example, for GAF with a graphene thickness of 84.2 nm, the measured length is 5 cm, the measured bulk resistance of GAF is 285 Ω, and the radius of AF is 0.212 mm. Through equation (1), the conductivity of GAF can be calculated:

$$\text{G=}=\frac{\text{L}}{\text{R×[π}\text{(r+t)}^{2}]}$$

$$= \frac{\text{5×}{10}^{-2}}{285\text{×[π×}{\text{(0.212×}{10}^{-3}\text{+}\text{84.2}\text{×}{10}^{-9}\text{)}}^{2}]}\approx1241 (S\cdot m^{-1})$$

Due to the radius of the AF (𝑟) of 0.212 mm being significantly greater than the graphene layer thickness ($\text{t}$), the cross-sectional area (S) can be approximated $\pi r^{2}$.

To validate the above analysis, in this study, the cross-sectional area S can be calculated by dividing the linear mass density (0.0041 g cm^-1^) by the material density of a single fiber (2.91 g cm^-3^).

For example, for GAF with a graphene thickness of 1.7 nm, the measured length is 5 cm, the measured bulk resistance of GAF is 8370 Ω, and the conductivity of GAF can be calculated as follows:

$$\text{G=}=\frac{\text{L}}{\text{R×S}}=\frac{\text{5×}{10}^{-2}}{\text{8370×0.14089×}{10}^{-6}}\approx42 (S\cdot m^{-1})$$

For example, for GAF with a graphene thickness of 84.2 nm, the measured length is 5 cm, the measured bulk resistance of GAF is 285 Ω, and the conductivity of GAF can be calculated as follows:

$$\text{G=}=\frac{\text{L}}{\text{R×S}}=\frac{\text{5×}{10}^{-2}}{\text{285×0.14089×}{10}^{-6}}\approx1245 (S\cdot m^{-1})$$

The measurement results of GAF conductivity are basically consistent with those calculated by equation (1).

[1] C. J. Shearer, A. D. Slattery, A. J. Stapleton, J. G. Shapter, C. T. Gibson, *Nanotechnology* **2016**, 27, 125704.
